# Renal Metabolome in Obese Mice Treated with Empagliflozin Suggests a Reduction in Cellular Respiration

**DOI:** 10.3390/biom12091176

**Published:** 2022-08-25

**Authors:** Surabhi Bangarbale, Blythe D. Shepard, Shivani Bansal, Meth M. Jayatilake, Ryan Kurtz, Moshe Levi, Carolyn M. Ecelbarger

**Affiliations:** 1 Department of Medicine, Georgetown University, Washington, DC 20057, USA; 2 Department of Human Science, Georgetown University, Washington, DC 20057, USA; 3 Proteomic & Metabolomics Shared Resource, Georgetown University, Washington, DC 20057, USA; 4 Department of Biochemistry and Molecular & Cellular Biology, Georgetown University, Washington, DC 20057, USA

**Keywords:** SGLT2, gluconeogenesis, oxidative phosphorylation, renal, proximal tubule

## Abstract

Sodium glucose cotransporter, type 2 inhibitors, such as Empagliflozin, are protective of the kidneys by unclear mechanisms. Our aim was to determine how Empagliflozin affected kidney cortical metabolome and lipidome in mice. Adult male TALLYHO mice (prone to obesity) were treated with a high-milk-fat diet, or this diet containing Empagliflozin (0.01%), for 8 weeks. Targeted and untargeted metabolomics and lipidomics were conducted on kidney cortex by liquid chromatography followed by tandem mass-spectroscopy. Metabolites were statistically analyzed by MetaboAnalyst 5.0, LipidSig (lipid species only) and/or CEU Mass Mediator (untargeted annotation). In general, volcano plotting revealed oppositely skewed patterns for targeted metabolites (primarily hydrophilic) and lipids (hydrophobic) in that polar metabolites showed a larger number of decreased species, while non-polar (lipids) had a greater number of increased species (>20% changed and/or raw *p*-value < 0.05). The top three pathways regulated by Empagliflozin were urea cycle, spermine/spermidine biosynthesis, and aspartate metabolism, with an amino acid network being highly affected, with 14 of 20 classic amino acids down-regulated. Out of 75 changed polar metabolites, only three were up-regulated, i.e., flavin mononucleotide (FMN), uridine, and ureidosuccinic acid. Both FMN and uridine have been shown to be protective of the kidney. Scrutiny of metabolites of glycolysis/gluconeogenesis/Krebs cycle revealed a 20–45% reduction in several species, including phosphoenolpyruvate (PEP), succinate, and malic acid. In contrast, although overall lipid quantity was not higher, several lipid species were increased by EMPA, including those of the classes, phosphatidic acids, phosphatidylcholines, and carnitines. Overall, these analyses suggest a protection from extensive metabolic load and the corresponding oxidative stress with EMPA in kidney. This may be in response to reduced energy demands of the proximal tubule as a result of inhibition of transport and/or differences in metabolic pools available for metabolism.

## 1. Introduction

The modern class of sodium glucose cotransporter, type 2 inhibitors (SGLT2i), including Empagliflozin (EMPA), were developed in the first decade of the 2000′s as a refinement of the original drug, Phlorizin (found in root bark, leaves, and apples), and shown to induce glycosuria [1]. Currently, four members of this class, “gliflozins”, are approved for treatment to reduce hyperglycemia in type 2 diabetes (T2D), i.e., EMPA, Canagliflozin, Dapagliflozin, and Ertugliflozin. A number of clinical studies, including Canigliflozin and Renal Events in Diabetes with Established Nephropathy Clinical Evaluation (CREDENCE) [2,3], DAPAgliflozin in patients with Chronic Kidney Disease (DAPA-CKD) [4], and EMPAgliflozin RandomizEd parallel Group cardiovascular OUTCOME (EMPA-REG OUTCOME) [5,6] have demonstrated the efficacy of these medications not only to reduce hyperglycemia, but, in many cases, to improve renal function and delay the onset of CKD in T2D.

The mechanism(s) whereby the gliflozin class of medications is protective of the kidney is not entirely known but likely multifactorial. Many of the benefits of SGLT2i on the kidneys may be indirect, i.e., generalized protection due to improvement in whole-body phenotype including weight loss, euglycemia, and reduced blood pressure; however, direct effects of the gliflozin class on the kidney cellular environment are also proposed [7]. Gliflozins have been demonstrated to alter cellular metabolism, inflammation, and fibrosis, at the level of the kidney, and in particular, in the proximal tubule, where glucose reabsorption is markedly reduced [8]. Metabolites are small organic intermediate or terminal products of molecular interactions between different proteins, signaling cascades, and cellular environments. Assessing the global footprint of the metabolic status of the kidneys can provide insight into the identity of protective mechanisms of these medications.

To elucidate protective actions of EMPA on the kidneys, we utilized untargeted and targeted metabolomics and lipidomics (LC-MS) on the kidney cortex (primarily proximal tubule cells) obtained from male TALLYHO/Jng mice (obese, insulin resistant, and pre-diabetic) [9] treated with a high-milk fat diet (pro-inflammatory) [10,11,12] plus/minus EMPA. Because T2D can cause significant metabolic changes in the kidneys, we decided to use an animal model of pre-diabetes (rather than frank T2D), with little pre-existing renal pathology, to evaluate early changes in metabolites that might underlie the physiology of the changes.

## 2. Materials and Methods

### 2.1. Study Design

Male TALLYHO/Jng (obese and insulin resistant) mice from our breeding colony (~4-months-old) were randomized to receive *ad libitum* access to control high-milk-fat diet (Research Diets, D08061904, New Brunswick, NJ, USA) or Empagliflozin (MedChem Express, Monmouth Junction, NJ, USA) incorporated into high-milk-fat diet by Research Diets@ 0.01% (by weight, *n* = 8/group). This provided about 10 mg/kg·bw/d EMPA, given our estimate of 4 g of chow consumed per day. Our dose was based on previous studies showing this dose effectively reduced insulin resistance and markers of non-alcoholic steatohepatitis (NASH) in diabetic mice [13]. Glucose was measured by dipsticks (UriScan™, Biosys Laboratories, South Pasadena, CA, USA) on spot urine in week 1 to ensure the drug was effective in causing glycosuria. Mice were weighed weekly. One treated mouse died prior to the end of the 8-weeks leaving (*n* = 8, control and *n* = 7, EMPA).

### 2.2. Tissue Collection

After 8 weeks of treatment, mice were euthanized under isoflurane anesthesia. A laporatomy was performed and blood was drawn from the heart into heparinized syringes. Blood was centrifuged at 1500× *g* to obtain plasma. The right and left kidneys were perfused through the heart with phosphate-buffered saline (PBS, 6 mL), removed, weighed, and then bisected coronally. The cortex was dissected away from the medulla and several homogenously minced cortex fractions were frozen (~100 mg each) at −80 °C. An additional fraction was solubilized fresh into Laemmli buffer for western blotting (see below).

### 2.3. Western Blotting

Kidney cortex homogenates were prepared into a sucrose/triethanolamine buffer containing protease inhibitors (HALT, ThermoFisher Scientific, Waltham, MA, USA) as we have previously described [14]. Western blots were conducted by loading and electrophoresing 20 μg protein in each lane of a BioRad (Hercules, CA, USA) 8–16% polyacrylamide gel, then blotting onto a 0.4 μm nitrocellulose membrane (BioRad). The membrane was incubated with 0.1% Ponceau Red (ThermoFisher Scientific) to stain all proteins to facilitate loading correction. After blocking in a 5% skimmed-milk solution, blots were probed with SGLT1 (NovusBio, NBP2-20338, Littleton, CO, USA) or SGLT2 (kind gift from Hermann Koepsell) [15,16] primary rabbit polyclonal antibodies both at 1:1000. After treating with anti-rabbit secondary coupled to horseradish peroxidase (ThermoFisher Scientific), blots were treated with chemiluminescence reagents (SuperSignal™ West Femto Maximum Sensitivity Substrate, ThermoFisher Scientific) and imaged either on film or with an Azure Biosystems C-Series Imager (Dublin, CA, USA).

### 2.4. Glucose Measurement

Glucose was measured using an Amplex™ Red Glucose/Glucose Oxidase Assay Kit (Invitrogen, Waltham, MA, USA) in plasma and in kidney cortex. A small piece of cortex was homogenized in PBS plus protease inhibitor (HALT), centrifuged at 10,000× *g*, and the supernatant retained for glucose determination. The protein concentration of the supernatant was determined by a bicinchoninic acid assay (BCA Protein Assay, ThermoFisher Scientific) and used to normalize kidney glucose levels.

### 2.5. Metabolite Analysis

We conducted both targeted and untargeted metabolomics in our Metabolomics Shared Resource (Georgetown University), as previously described [17]. For untargeted analyses, an Acquity UPLC system connected to an electrospray ion source coupled with a quadrupole time-of-flight mass spectrometer (ESI-Q-TOF, Xevo-G2S (Waters Corporation, Milford, MA, USA) operating in positive and negative ionization mode was used. The targeted approach allowed for quantitation of around 360 endogenous molecules using a QTRAP^®^ 5500 liquid chromatography tandem mass spectroscopy system (LC-MS/MS, Sciex, Framingham, MA, USA). Approximately 100 mg renal cortex sample was dissolved in 200 μL of extraction buffer (methanol/water 50/50) containing 200 ng/mL of debrisoquine (DBQ) as an internal standard for the positive mode and 200 ng/mL of 4-nitrobenzoic acid as an internal standard for the negative mode. The samples were vortexed for 30 s, incubated on ice for 20 min, then incubated at −20 °C for 20 min. Next, samples were centrifuged at 13,000 rpm for 20 min at 4 °C, and the supernatant obtained for analysis. Five microliters of the supernatant was injected onto a Kinetex 2.6 μm 100 Å 100 × 2.1 mm column (Phenomenex) using a SIL-30 AC auto sampler (Shimadzu, Kyoto, Japan) connected to a high-flow LC-30AD solvent-delivery unit (Shimadzu) and a CBM-20A communication bus module (Shimadzu) online with the QTRAP 5500 operating in both the positive and negative ion modes. A binary solvent comprised of water with 0.1% formic acid (solvent A) and acetonitrile with 0.1% formic acid (solvent B) was used. The extracted metabolites were resolved at a 0.2 mL/min flow rate starting with 100% solvent A and holding for 2.1 min. They next moved to 5% of solvent A, over a period of 12 min, holding for 1 min, then equilibrating to initial conditions over a period of 7 min using an auto sampler temperature 15 °C and an oven temperature of 30 °C. Source and gas settings for the method were as follows: curtain gas = 35, CAD gas = medium, ion spray voltage = 2500 V in positive mode and −4500 V in negative mode, temperature = 400 °C, nebulizing gas = 60, and heater gas = 70. The data were normalized to the internal-standard area and processed using MultiQuant 3.0.3 (Sciex). To ensure high quality and reproducibility of LC-MS data, a number of measures were taken. Quality control (QC) samples were injected initially, and then periodically (after every 20 samples) to monitor shifts in signal intensities and retention time. We also ran a National Institutes of Standards and Technology (NIST) plasma control sample (after every 20 samples), prepared in the same manner, to check instrumental variance. Blank solvent runs were conducted between sets of samples (after every 10 samples before and after pooled QC samples) to minimize carry-over effects.

### 2.6. Lipid Analysis

Minced cortex was homogenized in beaded tubes containing 100 μL of chilled isopropanol with added internal standards. The samples were next vortexed for 1 min and kept on ice for 30 min, then incubated at −20 °C for 2 h for complete protein precipitation. This was followed by centrifugation at 13,000 rpm for 20 min at 4 °C. The supernatant was transferred to a vial for LC-MS analysis. Five μL of each sample was injected onto an XBridge BEH amide column, 3.5 μm, 4.6 × 100 mm (Waters) using a SIL-30 AC auto sampler (Shimadzu) connected to a high flow LC-30AD solvent delivery unit (Shimadzu) and a CBM-20A communication bus module (Shimadzu) online with the QTRAP 5500 (Sciex) operating in both positive and negative ion mode. A binary solvent comprised of acetonitrile/water 95/5 as solvent A and acetonitrile/water 50/50 as solvent B both in 10 mM ammonium acetate was used for resolution. Lipids were resolved at a 0.7 mL/min flow rate. Initial gradient conditions started with 100% of solvent A, shifting towards 99.9% of solvent A, over 3 min, 94% of solvent A, over 3 min, and 25% of solvent A, over 4 min. Finally, we washed with 100% solvent B for 6 min and equilibrated to initial conditions (100% of solvent A) over 6 min using an auto sampler temperature 15 °C and oven temperature 35 °C. Source and gas settings were as follows: curtain gas = 30, CAD gas = medium, ion spray voltage = 5.5 kV in positive mode and −4.5 kV in negative mode, temperature = 550 °C, nebulizing gas = 50, and heater gas = 60. We measured 20 classes of lipid molecules, including diacylglycerols (DAG), cholesterol esters (CE), sphingomyelins (SM), phosphatidylcholines (PC), triacylglycerols (TAG), free fatty acids (FFA), ceramides (CE), dihydroceramides (DCER), hexosylceramides (HCER), lactosylceramides (LCER), phosphatidylethanolamines (PE), lysophosphatidylcholines (LPC), lysophosphatidylethanolamines (LPE), phosphatidic acids (PA), lysophosphatidic acids (LPA), phosphatidylinositols (PI), lysophosphatidylinositols (LPI), phosphatidylglycerols (PG), acylcarnitines (AC), and phosphatidylserines (PS).

### 2.7. Targeted Metabolomics

**Figure 1:**
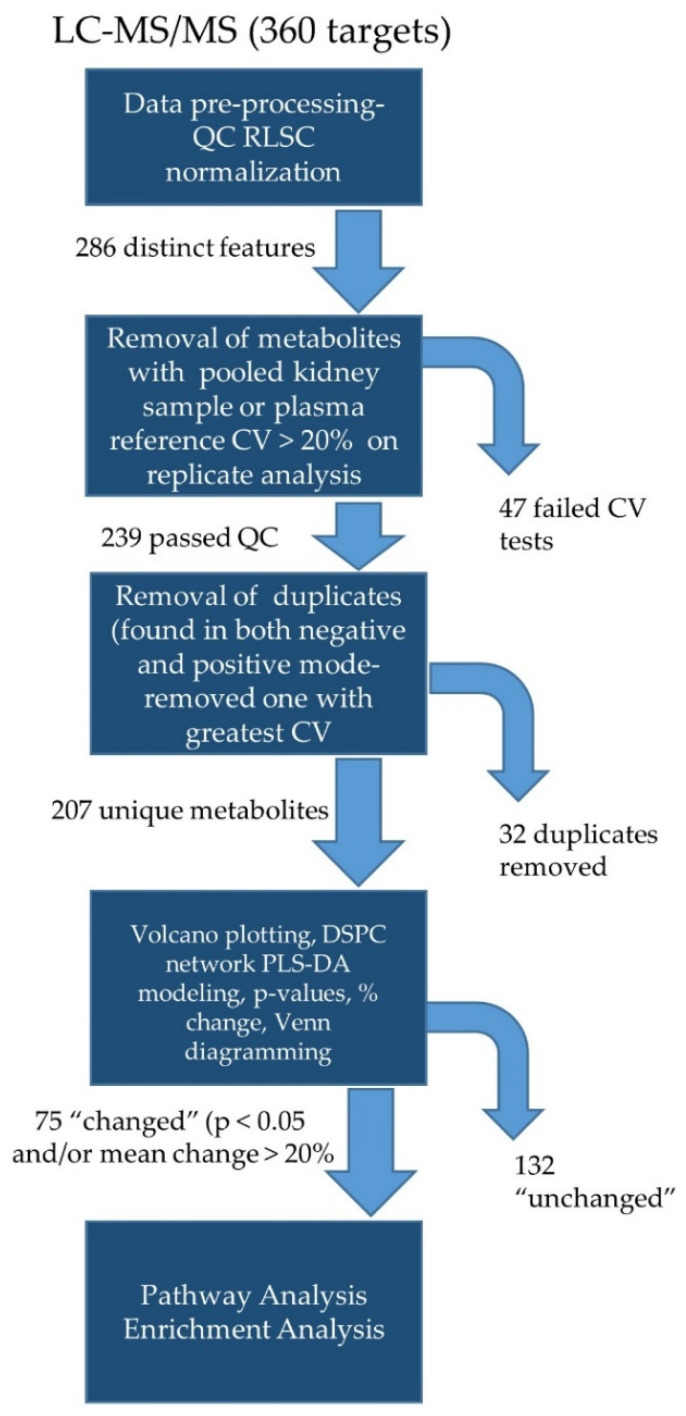
Workflow for targeted metabolomics. Peak pre-processing included quality-control-based robust LOESS signal correction (QC RLSC) for signal drift and batch correction. CV- coefficient of variation- used to determine stability of signal (>20% on replicates excluded). DSPC- debiased sparse partial correlation; PLS-DA- partial least squares differential analysis.

In the targeted approach (Figure 1), kidney samples were analyzed for a panel of 360 metabolites (Supplemental Table S1) in both the positive and negative modes. Manual peak quality was evaluated, and polar metabolites preprocessed using a signal-to-noise ratio > 20 and a retention time tolerance of 5 s. Two-hundred and eighty-six (286) reliable features (distinct peaks) were identified. Data for downstream analyses was further refined based on the coefficient of variation (CV) for each metabolite in the pooled kidney “quality control” (QC) samples, run at 20-sample intervals. Metabolites in the pooled samples with >20% CV were eliminated from downstream analyses. Two-hundred and seventy-four (274) peaks passed this quality check. Next, we screened out metabolites that showed >20% CV in QC plasma samples obtained from NIST. Thirty-five (35) metabolites failed this test and were eliminated. Another 26 metabolites were not detected in all plasma samples, but were found in the experimental kidney homogenates and thus were included. Of the remaining 239 metabolites, 32 were duplicates (detected in both positive and negative modes) and the data set was further shortened by selecting the duplicate with the lowest CV. Thus, 207 unique metabolites underwent bioinformatics analysis.

The entire data set (207 compounds, QC, RLSC normalized peak intensities) was entered into the MetaboAnalyst (Version 5.0, Xia Lab at McGill University, Montreal, QC, Canada). Statistical Analysis (one factor) Module as a CSV file. Log_10_ transformations and Pareto scaling was conducted to normalize data. Volcano plots were generated with parameter setting raw *p*-values < 0.05 and absolute fold change >1.2 (Log_2_ = ±0.26 or 20% change) considered significant. Hierarchical clustering was performed on the top 25 compounds (sorted by *p*-value) using autoscalable features, Euclidean (distance measure), and Ward (clustering feature) as radial choices. DSPC (debiased sparse partial correlation) networking and PLS-DA (partial least squares differential analysis) was also conducted. Seventy-five (75) metabolites with *p* < 0.05 and/or fold-change >1.2 were next entered into the Enrichment Analysis Modules (MetaboAnalyst) for deeper interrogation by probing the Small Molecule Pathway Database (SMPDB) [18] and Kyoto Encyclopedia of Genes and Genomes (KEGG).

### 2.8. Targeted Lipidomics

Lipid targets (1043) evaluated are shown in Supplemental Table S2. The workflow for targeted lipidomics (Supplemental Figure S1) was similar to that used for targeted metabolomics, including the use of MetaboAnalyst 5.0 for statistical computation and generation of volcano plots and hierarchical clustering. This was followed by additional analysis with LipidSig [19] to allow for input and integration of class, functional, and structural information to refine analysis of regulated lipid species.

### 2.9. Untargeted Analyses

The raw data files containing spectra were converted into Network Common Data (NetCDF) files for pre-processing. MetaboAnalyst 5.0 was used to further process spectra and calculate mass and retention time (mz_rt). The untargeted approach resulted in mass-to-charge (mz) ratios on 2706 analytes in the positive mode and 2656 analytes in the negative mode. Spectral intensities were further normalized by log_10_ transformation and Pareto scaling. Volcano plots were generated and *p* < 0.05 and/or log2 fold change >±0.26 were considered potentially “changed” and underwent further analysis. Mass-over-charge ratios for selected metabolites were putatively identified with a database search by applying the online version of CEU Mass Mediator (Universidad CEU, San Pablo, Brazil).

## 3. Results

### 3.1. EMPA Did Not Affect Weights but Lowered Plasma Glucose

No treatment differences were observed in the final body weight or kidney weight of the mice (Supplement Figure S2A–C). Both groups of mice gained about 10 g over 8 weeks. In the first week, glucosuria was confirmed in the EMPA-treated mice (and absent in the control mice) by dipstick (data not shown). Plasma glucose was reduced 51% (*p* < 0.02) in the treated mice (Figure S2D). We also measured glucose in the kidney cortical tissue but did not observe a significant difference (Figure S2E). Western blots of SGLT1 (found in proximal tubule S3 segment and not a target of EMPA) and SGLT2 (S1/S2 segments, EMPA target) were conducted on kidney cortex and showed a modest non-significant reduction in both proteins (Figure S2F) with EMPA.

### 3.2. Targeted Metabolites Down-Regulated by EMPA in Kidney Cortex

Of the 207 metabolites evaluated, 75 were filtered in for additional analysis of potentially regulated pathways using threshold inclusion of *p* < 0.05 and/or +/−20% (±0.26 log2 fold change). The top 10 reduced metabolites (ranked by percent of decrease) are shown in Table 1. Note, four metabolites did not have *p* < 0.05 due to high variability in the Control group. The top two (with a mean reduction over 50%) were carbamoyl phosphate and spermine.

**Figure 2:**
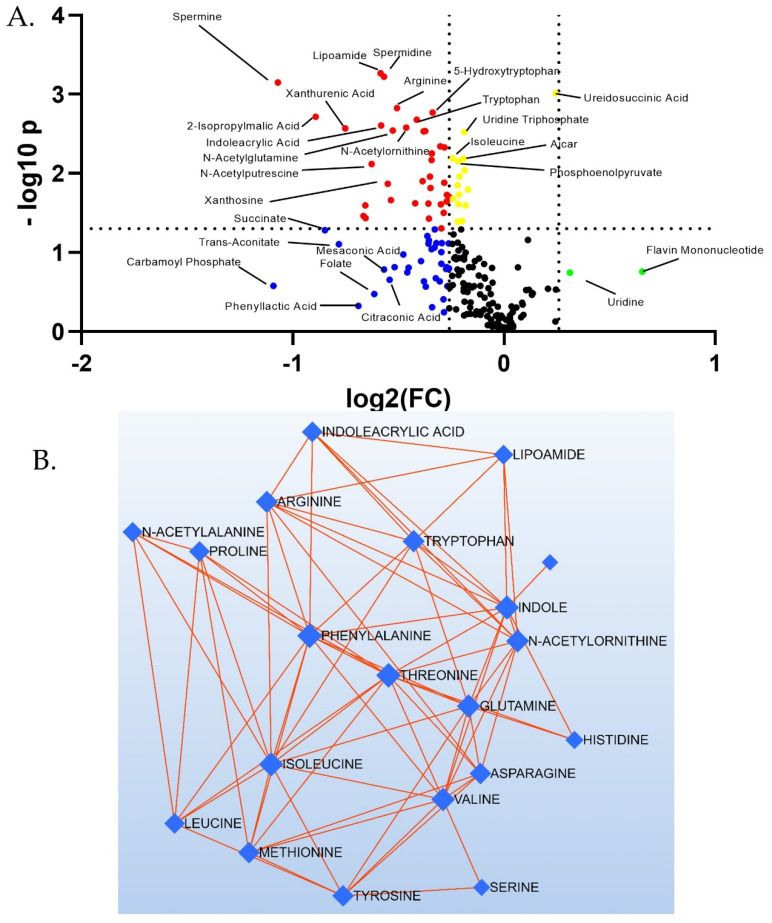
Targeted metabolomics reveals a greater number of down-regulated species. (**A**) Volcano plot of all 207 features: red dots—both *p* < 0.05 and log2(FC) < −0.26; yellow dots- *p* < 0.05 and log2(FC) > −0.26 and <+0.26; blue dots- *p* > 0.05 and log2(FC) < −0.26; black dots- *p* > 0.05 and log2(FC) > −0.26 and <+0.26; green dots- *p* > 0.05 and log2(FC) > +0.26. (**B**) Amino acid network showing the most highly regulated subnetwork. Red edges show positive correlations. The thicker the edge, the stronger the correlation.

A Volcano plot for all 207 metabolites illustrates the sharp discrimination toward overall reduction of targeted species with EMPA (Figure 2A). Species above the dotted horizontal line had *p* < 0.05, and those outside of the vertical dotted lines had log2(FC) > ±0.26. Thus, those features satisfying both criteria are above and external to the dotted lines (red dots). Note, no species were both increased over 1.2 fold and had *p* < 0.05. Those satisfying one but not the other are shown in yellow, blue, or green dots. Debiased Sparse Partial Correlation (DSPC) network modeling revealed a sub-network of amino acids containing 20 nodes and 73 edges. Nodes represent significantly changed (*p*-value) species and red edges show a positive correlation between metabolites in a network. All these amino acids were down-regulated in abundance in kidney cortex, with empagliflozin (Figure 2B).

**Figure 3:**
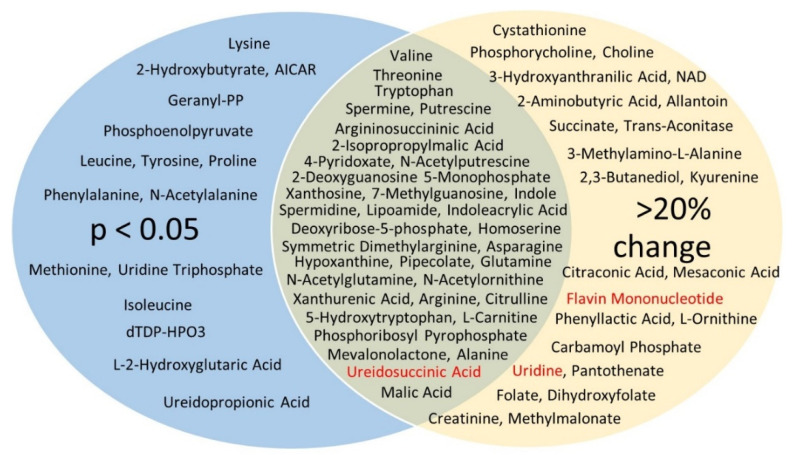
Venn diagram of changed targeted metabolites. In blue (16) are those in which *p* < 0.05 but were less than 20% changed; in yellow (24) are those that were greater than 20% changed, but *p* > 0.05; and the green overlap metabolites (35) satisfied both criteria. Those in red showed an increase with EMPA. All other (in black) were decreased.

**Figure 4:**
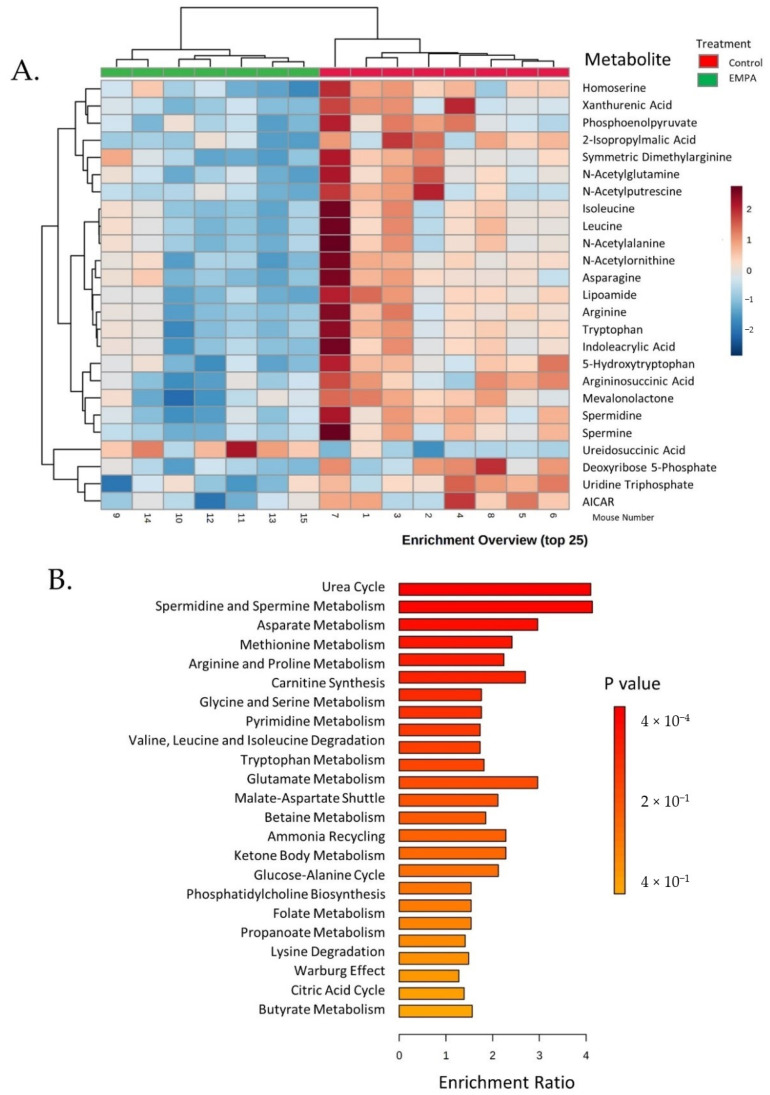
Clustering and enrichment of targeted metabolites. (**A**) Hierarchical clustering (top 25) of metabolite levels in EMPA (green, left) and control (red, right)-treated mice kidney cortex. Key shows relative levels of metabolites (log base 2) with color intensity scale red indicating high levels and blue low; (**B**) enrichment overview of main pathways down-regulated by EMPA.

The two-dimensional scores plot for the Partial Least Squares Discrimination Analysis (PLSDA) is shown in Supplement Figure S3A. Important features in Component 1 are shown in Figure S3B. The two most important features in Component 1 were carbamoylphosphate, followed by spermine. A Venn diagram was constructed displaying those metabolites (75 in total) with raw *p* < 0.05 and/or changes >20% (Figure 3). All but three (ureidosuccinic acid, flavin mononucleotide, and uridine) were decreased with EMPA. In summary, 24 metabolites showed a >20% change in abundance (*p* > 0.05), 16 had *p* < 0.05 (change < 20%), and 35 had both *p* < 0.05 and change >20%. Of the 35, 7 were classic amino acids, i.e., valine, alanine, arginine, asparagine, glutamine, tryptophan, and threonine. Hierarchical clustering of the 25 species with the lowest *p*-values is shown in Figure 4.Among these species were a number of amino acids, spermine, and phosphoenolpyruvate. Over-representation analysis (Enrichment Analysis Module) of the 75 “changed” metabolites revealed a number of significantly altered pathways with urea cycle, spermine/spermidine metabolism, and aspartate metabolism topping the list when sorted by *p*-value (Figure 4B). The top 10 regulated pathways are provided in Table 2.

**Figure 5:**
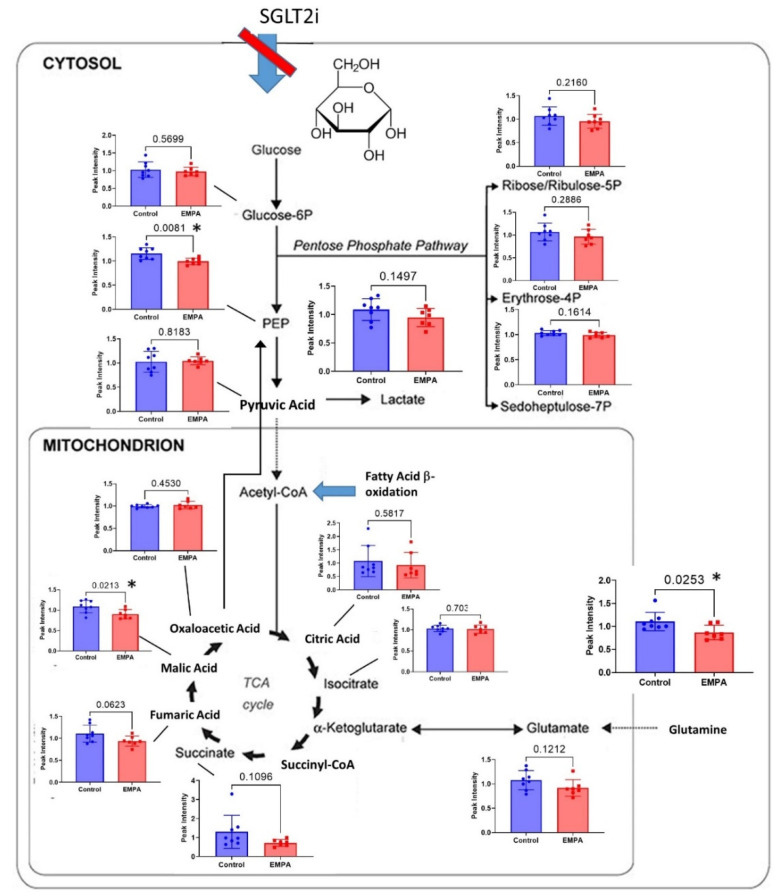
Metabolites involved in glycolysis and oxidative phosphorylation in general, were reduced with EMPA-Control (blue) and EMPA-treated (red) bars showing individual mouse values and mean (SEM) for metabolites evaluated in these pathways (*n* = 8 control, *n* = 7 EMPA); * indicates a significant difference by unpaired *t*-test (*p* < 0.05). Phosphoenolpyruvate (PEP), malic acid, and glutamine were down-regulated with *p* < 0.05. Succinate was reduced, on average, 45%.

We next evaluated metabolites of the glycolytic and oxidative phosphorylation pathways (Figure 5). No metabolites had EMPA/Control ratios greater than 1.0 in this pathway. Three had *p* < 0.05., i.e., glutamine, phosphoenolpyruvate (PEP), and malic acid. Another, i.e., succinate was reduced on average about 45% (4th highest reduction), suggesting an overall reduction in the activity of these energy producing and storing pathways with EMPA. 

### 3.3. Targeted Lipidomics Reveals a Number of Membrane Lipid Species Up-Regulated by Empagliflozin

**Figure 6:**
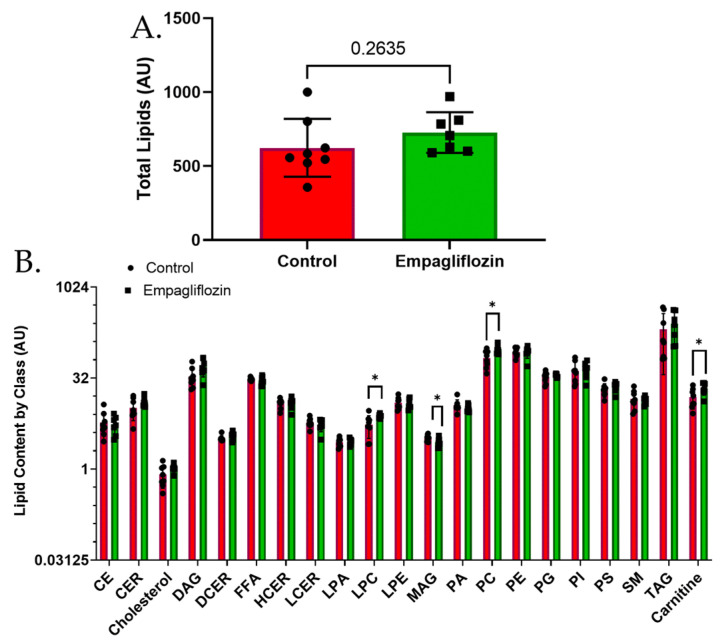
Overall abundance of lipids of various classes in kidney with EMPA. (**A**) Total lipid quantity was not significantly different. (**B**) Separation of lipid species into classes revealed increased levels of lysophosphocholines (LPC), phosphocholines (PC), and carnitines, while monoacylglycerols (MAG) were reduced; * indicates significantly different by unpaired *t*-test.

**Figure 7:**
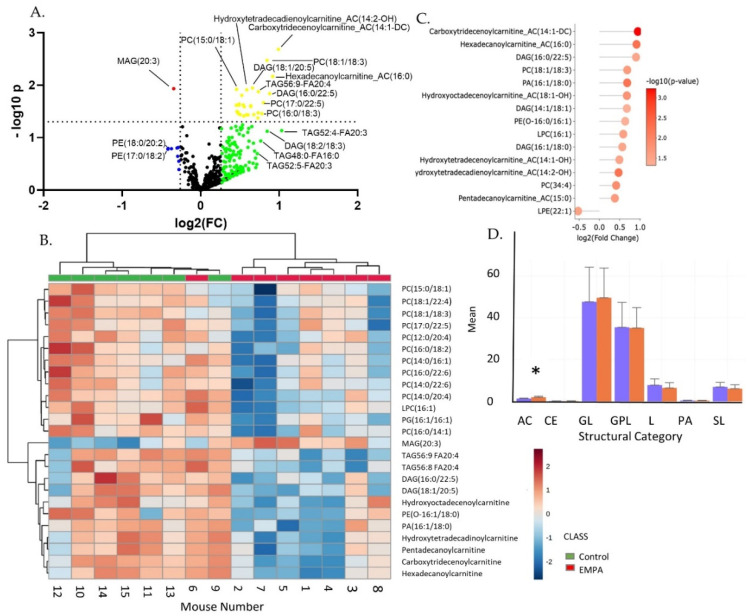
Targeted lipidomics reveals a number of up-regulated species. (**A**) Volcano plot; (**B**) hierarchical clustering (top 25) of metabolites (by *p*-value); key shows relative levels of metabolites (log2 fold change); (**C**) highly-regulated species; (**D**) structural features. Acyl carnitines (AC), as a structural category, were up-regulated (* *p* < 0.05, by unpaired *t*-test).

Targeted analysis of lipids revealed 448 distinct lipid-soluble features in which peak intensity was compared between the treated and untreated mice. Overall, the total abundance of lipid species in the kidney cortex was modestly increased (but not significantly changed) with EMPA (Figure 6A). Analysis of the specific classes revealed an increase in lysophosphocholines (LPC), phosphocholines (PC), and carnitines (CAR) with EMPA. Monoacylglycerols (MAG) were significantly decreased (Figure 6B). A volcano plot of all data is shown in Figure 7A. In contrast to metabolite analysis, lipidomics revealed a greater number of analytes were increased in the EMPA- versus control-treated mouse kidneys. Twenty-nine (29) analytes met the *p* < 0.05 threshold and over 50 were increased greater than 20% (Supplement Tables S3 and S4).

Of the top 50 features regulated by fold-change, 25 were of the class, triacylglycerol, i.e., storage lipids. In contrast, when sorted by *p*-value, the greatest “altered” lipids were phosphatidylcholines (PC) and phosphatidylethanolamines (PE), i.e., membrane lipids. Hierarchical clustering (Figure 7B) revealed consistent up-regulation of a large number of PC species with EMPA treatment. LipSig allowed for additional interpretation of those classes and predicted function of regulated species. Figure 7C shows those analytes with the lowest *p*-values relative to their log2 fold change. Carboxytridecenoylcarnitine (14:1) topped this list, along with five additional carnitine species. Figure 7D shows those lipids significantly regulated as a class. Because under-activity of the TCA cycle and changes in the lipid profile may affect oxidative stress, we compiled a list of metabolites (12) related to oxidative stress and how they were regulated by EMPA (Table 3).

### 3.4. Untargeted Metabolite Analyses

**Figure 8:**
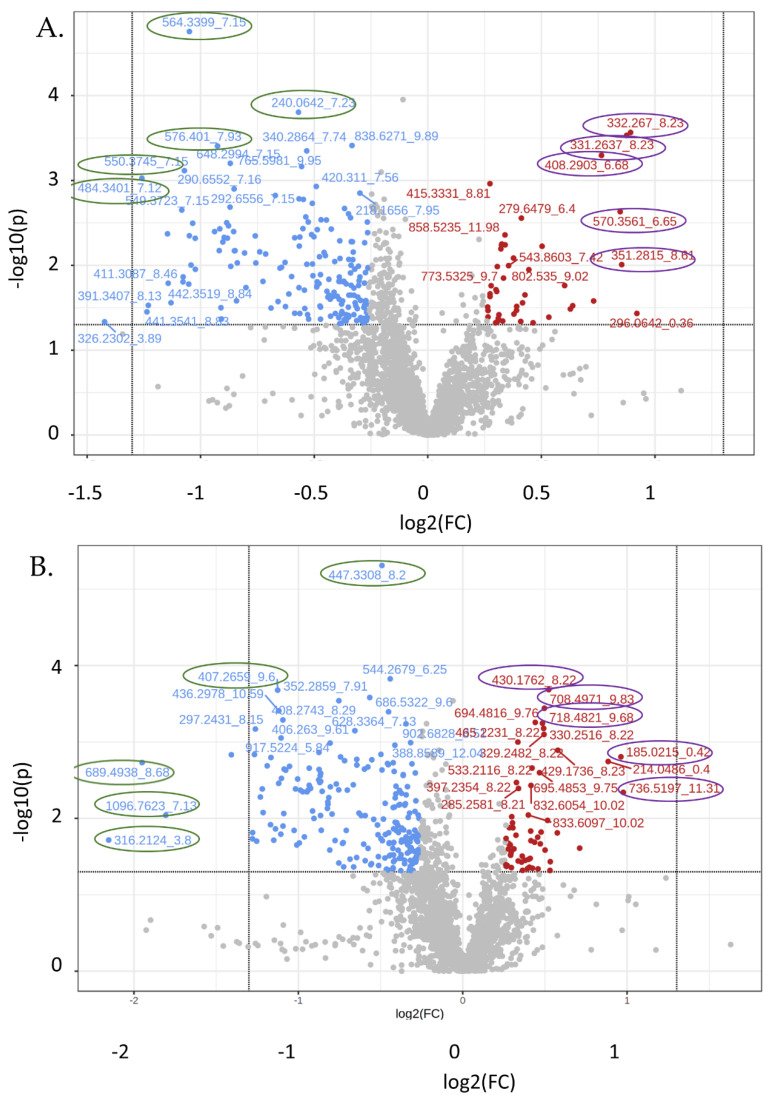
Volcano plot of untargeted features (mz_rt). (**A**) Positive; (**B**) negative modes. Blue and red dots indicate features changed (raw *p*-value < 0.05 and log2 FC > ±0.26). Encircled mz_rt were entered into CEU-Mass Mediator for annotation query.

Untargeted metabolomics revealed 2727 features in the positive mode and 2656 in the negative mode. Volcano plots (Figure 8) were skewed to the upper left in that a greater number of species were reduced in the kidney from EMPA-treated mice. Hierarchical clustering (Supplement Figure S4) provides a graphic demonstration of the most significantly changed mass-to-charge ratios. The 50 most highly changed species found in the positive mode by fold-change and by *p*-value are shown in Supplemental Tables S5 and S6, respectively. Similar tables for species found in the negative mode are in Supplemental Tables S7 and S8. From the volcano plots, we chose 10 relative “outliers” (showing extremes in *p*-values and fold change) in both the positive and negative modes (see Figure 8, circled mz_rt) to interrogate further using CEU Mass Mediator [20,21], an online metabolite annotation tool (Table 4). While a number of mz_rt had over 10 possible matches, for 2 spectra, 564.3399_7.15 and 407.2659_9.6, there was just one match. For the former, CEU Mass Mediator predicted 1-(2-methoxy-eicosanyl)-sn-glycero-3-phosphoethanolamine, and for the latter, Sinapoylspermine. Both species were reduced by EMPA treatment. In agreement with the targeted analyses, nearly all of the increased species were predicted to be modified phospholipids, e.g., phosphatidylcholine, phosphatidic acid, or phosphatidylethanolamine.

## 4. Discussion

SGLT2is have been proposed to have a number of protective actions on the kidney to combat progression to diabetic nephropathy [7,8,22,23,24]. SGLT2i are known to reduce GFR at the whole organ level by increasing the sodium load to the macula densa and activating tubuloglomerular feedback to constrict the renal afferent artery [16,25,26,27,28]. This in itself may result in long-term protection of the kidneys by reducing the work required to reabsorb sodium and glucose in the proximal tubule. Nonetheless, little is clearly understood regarding the effects of these agents on proximal tubule metabolism, the site at which these inhibitors act.

Integration of metabolomics with lipidomics provides a comprehensive understanding of metabolism at the cellular level, which is difficult to achieve with one platform alone [29]. Our findings may be broadly summarized in that the majority (>90%) of targeted species in the list of metabolites (primarily hydrophilic species) were down-regulated in kidney cortical cells, if changed at all, with Empagliflozin, while lipid species (hydrophilic) were primarily increased. This fundamental change in the balance of substrate types available for oxidation and anabolism under this state may have a role in the protective actions of this class of medications against renal disease. Here we discuss potential implications for our main findings in the context of kidney health.

We found EMPA reduced renal levels of a number of metabolic substances in the glycolytic/tricarboxylic acid (TCA)/gluconeogenic pathways, including malic acid, phosphenolpyruvate, and succinate. Many others, even if they did not quite reach statistical significance, were reduced by about 15%. These are the sole energy (ATP) generating pathways in any cell whether the energy comes from carbohydrate, fat, or protein. 

This may be a normal physiological response to reduced requirements for ATP due to an overall reduction in transepithelial transport and a reduction in Na + K + ATPase activity. SGLT2 inhibition would lessen the amount of sodium that accompanies glucose into the cell across the apical membrane. Thus, less ATP is required to pump the sodium back into the circulation, against the concentration gradient. Relatively higher ATP levels could be sensed by the cell, e.g., via adenosine monophosphate-activated protein kinase (AMPK), tuning down catabolic pathways [30]. Additional study of these specific pathways and the ratio of AMP-to-ATP would need to be performed to clarify whether this is indeed the case.

However, alternatively, the reduction in metabolites of the TCA/glycolysis/gluconeogenic pathways may result from EMPA directly inhibiting brush border reabsorption of these fuel sources (in addition to glucose), e.g., amino acids and fatty acids for oxidation. For example, gliflozins have been shown to reduce the activity of the sodium hydrogen exchanger, type 3 (NHE3) [31], in addition to their well-known action to reduce SGLT2 transport. In support of a reduction in need for ATP (rather than reduction in available substrate) is the fact that there was a general 50–100% increase in the levels of many storage forms of energy in the PT, i.e., triacylglycerides and diacylglycerols. This would suggest a reduction in energy utilization (cellular respiration) and an increase in energy storage. Also, in support of this is the fact that the water-soluble metabolites, at least in kidney, are not commonly storage forms of energy. The kidney does not store glycogen (water soluble) except under pathological conditions [32]. Thus, the fact that there was a generalized reduction in the water-soluble metabolites (primarily amino acids and carbohydrate derivatives) would suggest reduced flux through catabolic pathways (energy mobilizing).

A recent study showed proximal tubule (PT) makes up about 60% of the volume density of the renal cortex in male mice [33]. This cell type, at least under healthy conditions, relies primarily on oxidation of fatty acids for ATP generation [34,35]. Non-esterified fatty acids can be reabsorbed from the filtrate (some rely on sodium) or enter via the basolateral membrane. In general, the levels of FFA were not changed to any extent by EMPA to make up for the reduction in amino acids and other 2–6 carbon species, again supporting a reduced need for ATP.

In our targeted metabolomics analysis, amino acids (AA) reflected the largest chemical class of down-regulated species. Fourteen of the twenty common amino acids were reduced by EMPA. Pathway analysis using KEGG annotations revealed five of the six total pathways with a false discovery rate < 0.05 were related to amino acid biosynthesis or metabolism. AA are used for protein synthesis, acid-base regulation, or enter the TCA cycle at a number of points to be used for ATP-generation, glucose production, or triacylglycerol production. AA are reabsorbed from the PT lumen by regulated transport processes, including the sodium-dependent solute carrier transporters (SLC) [36]. AA may also be transported across the basolateral membrane, e.g., glutamine enters or exits (based on cellular need) via Slc38a3 (SNAT3). Cellular glutamine levels were approximately 20% reduced (*p* < 0.03) by EMPA. Glutamine is the most highly abundant free AA in plasma and in the body as a whole, and has traditionally been seen as a nitrogen carrier between organs [37]. A reduction in the levels of glutamine supports the concept of a tuned-down metabolism in the PT with EMPA.

In addition, many of the components of the urea cycle were reduced in kidney by EMPA treatment, e.g., carbamoyl phosphate, arginininosuccinate, arginine, and ornithine. The urea cycle is initiated by the conversion of glutamine to glutamate or the condensation of aspartate with citrulline to produce argininosuccinate. This cycle is classically studied in the liver; however, the proximal tubule of the kidney also expresses the enzymes involved in urea synthesis from ammonia. Down-regulation of nearly all cycle components likely suggests less ammonia production in PT. The urea cycle is tied to polyamine synthesis in that putrescine is produced from ornithine (after liberation of urea). This pathway also liberates fumarate, which feeds into the TCA cycle.

Down-regulation of the polyamine metabolism pathways extended to reduced spermine, spermidine, putrescine, and N-acetyl-putrescine. In fact, spermine was reduced by over 50% in the kidney tissue from the EMPA-treated mice. Polyamines have a number of essential functions, including in the very fundamental regulation of the aminoacyl-tRNA biosynthetic pathway (a highly down-regulated pathway), protein synthesis, and cell proliferation. In fact, members of this class have been shown to have anti-oxidant and anti-aging properties [38]. In a healthy kidney, polyamine levels in the kidney are tightly controlled. Polyamine depletion (spermine and spermidine) of HeLA cells by over-expression of spermidine/spermine N1-acetyltransferase 1 (SAT1) has been shown to reduce translation initiation, resulting in G1 arrest [39] and a senescence-like phenotype [40]. However, polyamines are also released during periods of cell stress or DNA damage. Moreover, excessive polyamine content is associated with toxicity in kidney, liver, and other organs [41]. Although the TALLYHO/Jng mice do not exhibit severe renal disease or fibrosis, it is possible that a reduction in this pathway is the result of less DNA damage in our high-fat fed obese mice.

There were only three metabolites (out of 75 changed) significantly up-regulated by EMPA, i.e., ureidosuccinic acid, flavin mononucleotide (FMN) and uridine. Ureidosuccinic acid (carbamyl-L-asparate) and uridine are both involved in pyrimidine biosynthetic pathways (as is carbamoyl phosphate). Thus, the increase in two components of this pathway suggests EMPA alters pyrimidine synthesis. Ureidosuccinic acid is produced from carbamoyl phosphate and aspartate. This compound is not well studied in kidney, and the approximate 18% increase is especially intriguing since carbamoyl phosphate levels (its precursor) were reduced by nearly 40%. Uridine is a pyrimidine nucleoside composed of uracil and ribose. It levels in the circulation have been shown to be up-regulated by fasting (through adipocyte production) [42]. Uridine has also been shown to prevent fatty liver in various mouse models [43,44]. Uracil (product of uridine by recycling) levels were not different, while uridine 5′ triphosphate (UTP) was significantly reduced. Kidney is primarily responsible for uridine excretion; thus, it is possible that the elevated renal cortical uridine in the EMPA-treated mice represented a separate pool reabsorbed or secreted from the blood.

FMN is a redox coenzyme used in energy metabolism and a product of riboflavin metabolism. FMN therapy has been shown to ameliorate oxidative stress and DNA damage in diabetic mice [45]. There are a number of studies showing protective and anti-oxidative effects of riboflavin [46]. Whether FMN levels are preserved in PT of the EMPA-treated mice due to less overall metabolism or for another reason, the relatively higher level is likely a protective sign.

Lipidomics is a newer class of “omics”, which has developed as a distinct field out of necessity due to the increasing complexity and diversity of this class of chemicals. In mammals, there are essentially eight major categories of lipids, including fatty acyls, glycerolipids, glycerophospholipids, sterol lipids, prenol lipids, sphingolipids, saccharolipids, and polyketides [47]. We found EMPA led to a general increase in lipids, in renal cortex, in particular glycerophospholipids (GL) of certain classes, which are the major component of biologic membranes. Various species of phosphatidylcholine (PC) constituted 12 of the 25 mostly highly up-regulated species (based on *p*-values/hierarchical clustering, Figure **7**). The ratio of PC to PE in the membrane has been associated with membrane integrity, at least in the liver, and this ratio was found to be decreased in steatohepatitis in mice and in human patients with nonalcoholic steatohepatitis (NASH) [48]. PC are primarily localized to the outer leaflet of the mitochondrial membrane and PE within the inner leaflet. Other studies have shown greater activity of PE N-methyltransferase (PEMT), which facilitates conversion of PE to PC is protective of membrane integrity. Thus, there is the possibility that SGLT2i preserves PEMT activity in kidney cells in these high-fat fed mice. The sole class of lipids to be decreased by EMPA was monoacylglycerols (MAG). Additional study of the potential impact of this decrease is warranted.

Furthermore, a number of carnitine derivatives were increased with EMPA. A major function of carnitines is to transport long chain fatty acids (FA) into the mitochondria for β-oxidation. This would not be surprising with EMPA, given the reduction in availability of glucose in proximal tubule for glycolysis, lactate production, and eventual Krebs cycle generation of energy species for ATP generation. The PT primarily uses fatty acids for energy rather than glucose due to low-to-non-existent levels of hexokinase, the first enzyme in glucose metabolism. Thus, an increase in FA oxidation with EMPA in PT may not be readily observed. Nonetheless, other cells of the cortex (thick ascending limb) do use glucose and would be included in a whole cortex homogenate. Moreover, it is possible that circulating glucose is marginally reduced with EMPA, and thus there is greater β-oxidation in these other cell types, which leads to up-regulation of carnitine species.

With regard to the overall pattern of change, a study by Mulder et al. [49] examined plasma levels of metabolites and lipids in human patients treated with dapagliflozin. They observed, in general, somewhat opposite findings in that several amino-acid related metabolites, e.g., alanine and aspartate metabolism, histidine metabolism, and arginine and proline metabolism were increased by dapagliflozin treatment, while lipids, e.g., fatty acid branched pathway and fatty acid dicarboxylate, were decreased. The discrepancy between our study and theirs most likely relates to changes observed in the circulation versus in the kidney cells themselves.

## 5. Conclusions

Chronic Empagliflozin treatment of mice to inhibit SGLT2 leads to a generalized reduction in polar metabolites in the kidney cortex, including several amino acids, TCA cycle metabolites, and degradation products such as urea. In contrast, lipids, including membranous phospholipid species, in particular phosphatidylcholine, were increased. Overall, this may suggest a reduction in cellular requirements for or utilization of ATP (respiration), with concomitant greater energy, i.e., lipid storage. As a whole, this may be protective of the overall health of the kidney, but additional studies will be needed to flesh out the mechanisms.

## Figures and Tables

**Table 1:** Metabolites ^†^ down-regulated by empagliflozin in kidney (10 highest).

Compound Name	Control *	EMPA	*p*-Value ^‡^	EMPA-to-Control Ratio
Carbamoyl phosphate	1.38 ± 0.53	0.65 ± 0.14	0.23	0.47
Spermine	1.38 ± 0.18	0.66 ± 0.07	0.0036	0.48
2-Isopropylmalic acid	1.27 ± 0.12	0.68 ± 0.08	0.0021	0.54
Succinate	1.30 ± 0.31	0.72 ± 0.07	0.11	0.56
Trans-Aconitate	1.23 ± 0.27	0.71 ± 0.12	0.12	0.58
Xanthurenic acid	1.29 ± 0.14	0.77 ± 0.06	0.0057	0.59
Phenyllactic acid	1.10 ± 0.43	0.68 ± 0.08	0.39	0.62
2-Deoxyguanosine 5-monophosphate	1.33 ± 0.17	0.84 ± 0.11	0.036	0.63
4-Pyridoxate	1.27 ± 0.17	0.76 ± 0.10	0.022	0.63
Putrescine	1.22 ± 0.14	0.77 ± 0.08	0.018	0.64
Spermidine	1.24 ± 0.07	0.84 ± 0.06	0.00078	0.67

^†^ QC-RLSC normalized peak intensities, * mean ± sem (*n* = 8-control; 7-empagliflozin); ^‡^ unpaired *t*-test.

**Table 2:** Effect of Empagliflozin on discrete metabolite sets * in kidney (top 10 listed).

Metabolite Set	Total	Hits	Expect ^†^	*p*-Value ^‡^	Holm’s *p* ^¥^	FDR ^€^
Urea cycle	29	8	1.95	0.0004	0.038	0.038
Spermidine and spermine biosynthesis	18	5	1.21	0.0052	0.502	0.223
Aspartate metabolism	35	7	2.36	0.0068	0.66	0.22
Methionine metabolism	43	7	2.90	0.021	1.0	0.42
Arginine and proline metabolism	53	8	3.57	0.022	1.0	0.42
Carnitine synthesis	22	4	1.48	0.055	1.0	0.90
Glycine and serine metabolism	59	7	3.98	0.094	1.0	0.94
Pyrimidine metabolism	59	7	3.98	0.094	1.0	0.94
Valine, leucine, and isoleucine degradation	60	7	4.04	0.10	1.0	0.94
Trypotophan metabolism	60	7	4.04	0.10	1.0	0.94

* Entry included all (75 total) metabolites (from targeted analysis) with *p* < 0.05, (unpaired *t*-test) and/or a change >20% as determined by Enrichment Analysis (MetaboAnalyst 5.0); ^†^ based on chance; ^‡^ students *t*-test; ^¥^ Holmes-Bonferroni method to account for multiple comparisons; ^€^ FDR- false discovery rate—alternative to Bonferroni and controls for a low proportion of false positives (guards against type 1 errors).

**Table 3:** Oxidative-Stress-Related Metabolic and Lipidomic Species ^†^.

Compound Name	Control	EMPA	*p*-Value	EMPA-to Control Ratio	Function
Symmetric Dimethylarginine	1.16 ± 0.052	0.91 ± 0.059	0.0082	0.79	Inhibitor of arginine transport; marker of GFR
Lipoic Acid	0.87 ± 0.085	0.78 ± 0.11	0.52	0.90	Anti-oxidant
AICAR	1.08 ± 0.031	0.95 ± 0.027	0.0062	0.88	AMPK activator, anti-oxidant
Nicotinamide Adenine Dinucleotide	1.14 ± 0.12	0.83 ± 0.16	0.15	0.73	Co-Factor in Redox Reactions
Pyruvic Acid	1.02 ± 0.077	0.94 ± 0.071	0.40	0.89	Component of Glycolysis; anti-oxidant
Sarcosine	1.06 ± 0.079	0.90 ± 0.055	0.13	0.85	Derivative of glycine; causes oxidative stress
Uridine	0.93 ± 0.11	1.16 ± 0.098	0.16	1.24	Glycosylated pyrimidine; anti-oxidant
Flavin Mononucleotide	69 ± 0.046	1.08 ± 0.25	0.11	1.57	Produced from riboflavin; prosthetic group of oxidoreductases
Carboxytridecanoylcarnitine ^‡^	0.61 ± 0.11	1.21 ± 0.10	0.0021	1.99	Fat transport into mitochondria
Hexadecanoylcarnitine ^‡^	0.62 ± 0.12	1.17 ± 0.12	0.0068	1.88	Fat transport into mitochondria
Pentadecanoylcarnitine ^‡^	0.83 ± 0.05	1.16 ± 0.089	0.024	1.40	Fat transport into mitochondria
Hydroxyoctadecenoylcarnitine ^‡^	0.65 ± 0.12	1.04 ± 0.12	0.037	1.59	Fat transport into mitochondria

^†^ Sampling, not exhaustive; ^‡^ more than 10 modified carnitines were significantly increased by EMPA; in general carnitines can protect against oxidative stress the modifications are not well understood with regard to function.

**Table 4:** Metabolite Theoretical Annotation in Untargeted Species ^‡^.

Mz_rt *	FC	*p*-Value	Direction	Annotation †
Positive				
332.3_8.23	1.86	0.000272	Increased	3; modified PA species
331.3_8.23	1.83	0.000294	Increased	>10; modified eicosadienoic acid species
351.3_8.61	1.81	0.009813	Increased	6; modified SM or PE-ceramide
570.3_8.61	1.80	0.002333	Increased	>10; modified, LPC, CL, PC
408.3_6.68	1.70	0.000506	Increased	>10; Radarin & PG modified species
484.3_7.12	0.42	0.000941	Decreased	5; modified PA, taurine, sulfonic acid
550.3_7.15	0.48	0.000766	Decreased	2, modified vitamin D3 or lactone
564.3_7.15	0.48	1.76 × 10^−5^	Decreased	1; 1-(2-methoxy-eicosanyl)-sn-glycero-3-phosphoethanolamine; M + K
576.4_7.93	0.53	0.000393	Decreased	>10; PE or PC modifications
240.1_7.23	0.67	0.000157	Decreased	>10; carboxylic acid, gluco, & galactoside
**Negative**				
736.5_11.3	1.97	0.004575	Increased	>10; PE or PC modifications
185.0_0.42	1.95	0.001566	Increased	Modified carbohydrate, e.g., ribulose
430.2_8.22	1.44	0.000207	Increased	>10; tripeptide
708.5_9.83	1.41	0.000362	Increased	>10; PE or PC modifications
718.5_9.68	1.40	0.00057	Increased	>10; PE modifications
316.2_3.8	0.22	0.019235	Decreased	2; 3-hydroxynonanoyl carnitine and Tridecanoylglycine
689.5_8.68	0.26	0.001863	Decreased	>10; DG, PE, or PA modified species
1096.8_7.13	0.29	0.009105	Decreased	Nothing found
407.3_9.6	0.46	0.00021	Decreased	1; Sinapoylspermine
447.3_8.2	0.71	4.91 × 10^−6^	Decreased	9; vitamin K, quinone; cholesterol

^‡^ Selected 10 outliers on volcano plot for each mode; * Mass-to-charge ratio underscore retention time; † number of annotated species and their categories; positive mode potential adducts—M + H, M + 2H, M + 2H, M + Na, M + K, M + NH_4_, M + H-H_2_O; PA—phosphatidic acid; SM—sphingomyelin; PE—phosphoethanolamine; LPC—lysophosphatidycholine; CL—cardiolipin; PC—phosphatidylcholine; PG—phosphoglycerol; DG—diacylglycerol.

## Data Availability

Data in addition to what is found it the Results and Supplementary Data Section may be viewed by directly contacting the Corresponding PI, Carolyn Ecelbarger, ecelbarc@georgetown.edu.

## Supplementary Materials

The following are available online at https://www.mdpi.com/article/10.3390/biom12091176/s1, Figure S1: Workflow for targeted lipidomics, Figure S2: Effects of empagliflozin physiologic parameters, Figure S3: Partial Least Squares Discrimination Analysis (PLS-DA); Figure S4: Heatmap of highly changed untargeted metatabolites; Table S1: Targeted Metabolites; Table S2: Targeted Lipids; Table S3: Targeted Lipidomics Most Highly Changed (by Fold Change); Table S4: Targeted Lipidomics Most Highly Changed (by *p*-value); Table S5: Positive Mode Untargeted Most Highly Changed Mass-to-Charge (by Fold Change); Table S6: Positive Mode Untargeted Most Highly Changed Mass-to-Charge (by *t*-test); Table S7: Negative Mode Untargeted Most Highly Changed Mass-to-Charge (by Fold Change); Table S8: Negative Mode Untargeted Most Highly Changed Mass-to-Charge (by *t*-test).
