# Supplementary material for: Renal Metabolome in Obese Mice Treated with Empagliflozin Suggests a Reduction in Cellular Respiration
Source: Biomolecules. 2022 Aug 25;12(9):1176. doi: 10.3390/biom12091176 (PMC9496198; doi:10.3390/biom12091176)
Supplement: Supplementary file 1 [file biomolecules-12-01176-s001.zip › biomolecules-1728286-supplementary.pdf]

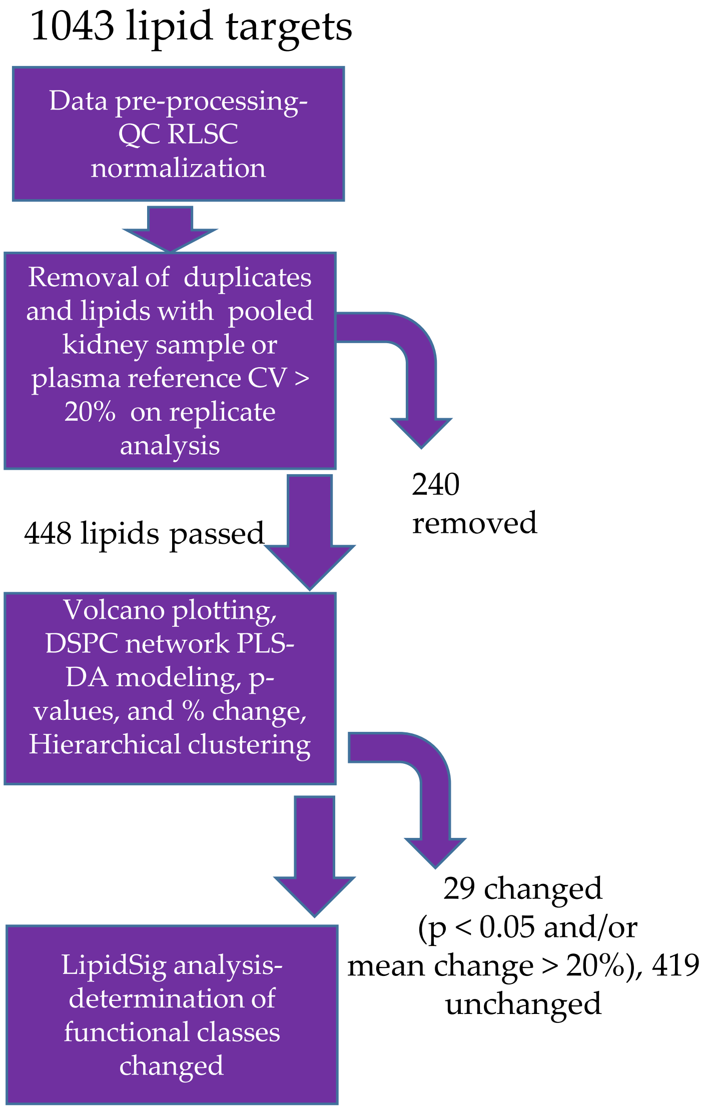

Figure S1- Workflow for targeted lipidomics- Peak pre-processing including quality-control-based robust LOESS signal correction (QC-RLSC) for signal drift and batch correction. CV- coefficient of variation- used to determine stability of signal (> 20% on replicates) excluded. DSPC- debiased sparse partial correlation; PLS-DA- partial least squares differential analysis squares differential analysis.

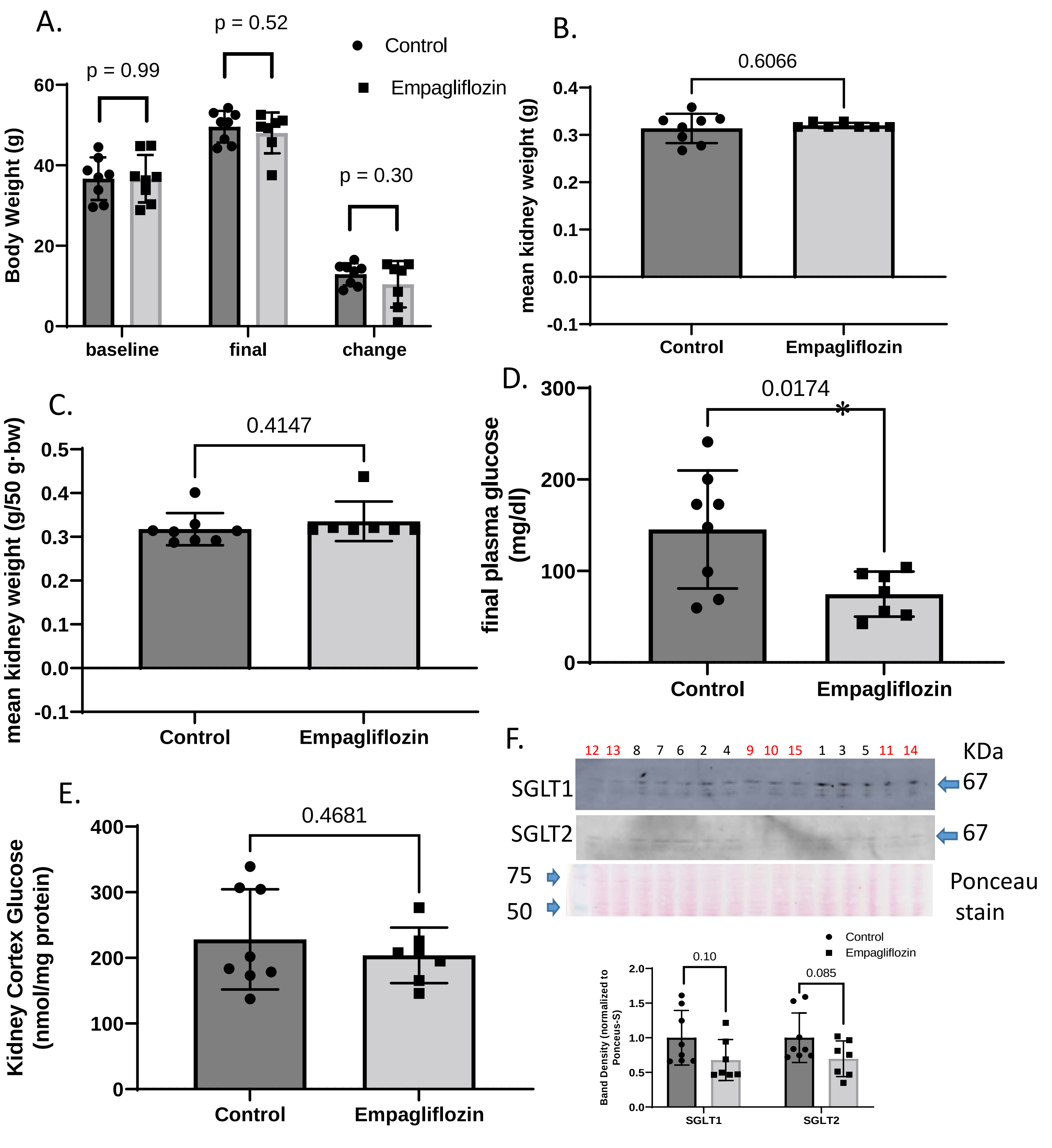

Figure S2- Effects of empagliflozin physiologic parameters- A. body weight and weight change; B. final absolute kidney weight; C. final body weight normalized kidney weights; D. final plasma glucose; E. kidney cortex glucose; F. Western blot of kidney cortex probed for SGLT1 and SGLT2 proteins (numbers above indicate sample, black- control, red- EMPA; Ponceau-stain-normalized band densities below. Data analyzed by unpaired t-test (n = 8 control, 7 empagllflozin). P-values are provided above bars, $p < 0.05$ considered significant.

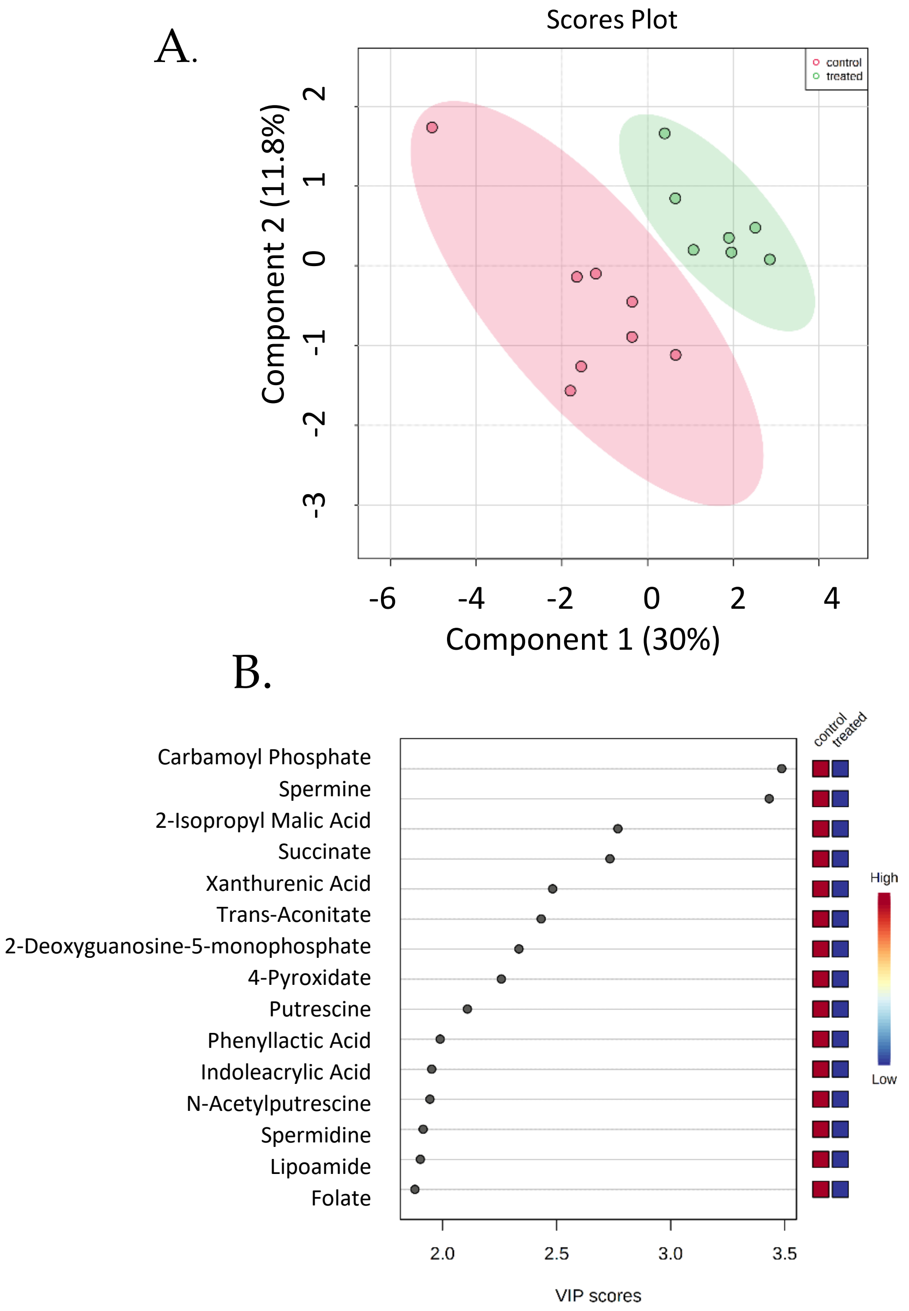

Figure S3- Partial Least Squares Discrimination Analysis (PLS-DA)- A. 2D-Scores Plot (explained variance for X) of important features of Component 1 versus 2 in analysis of metabolite data (MetaboAnalyst 5.0). Red- control; Green- treated. B. Important features of the PLS-DA; intensity scale on right indicates relative concentrations of the corresponding metabolite in each group.

A.

B.

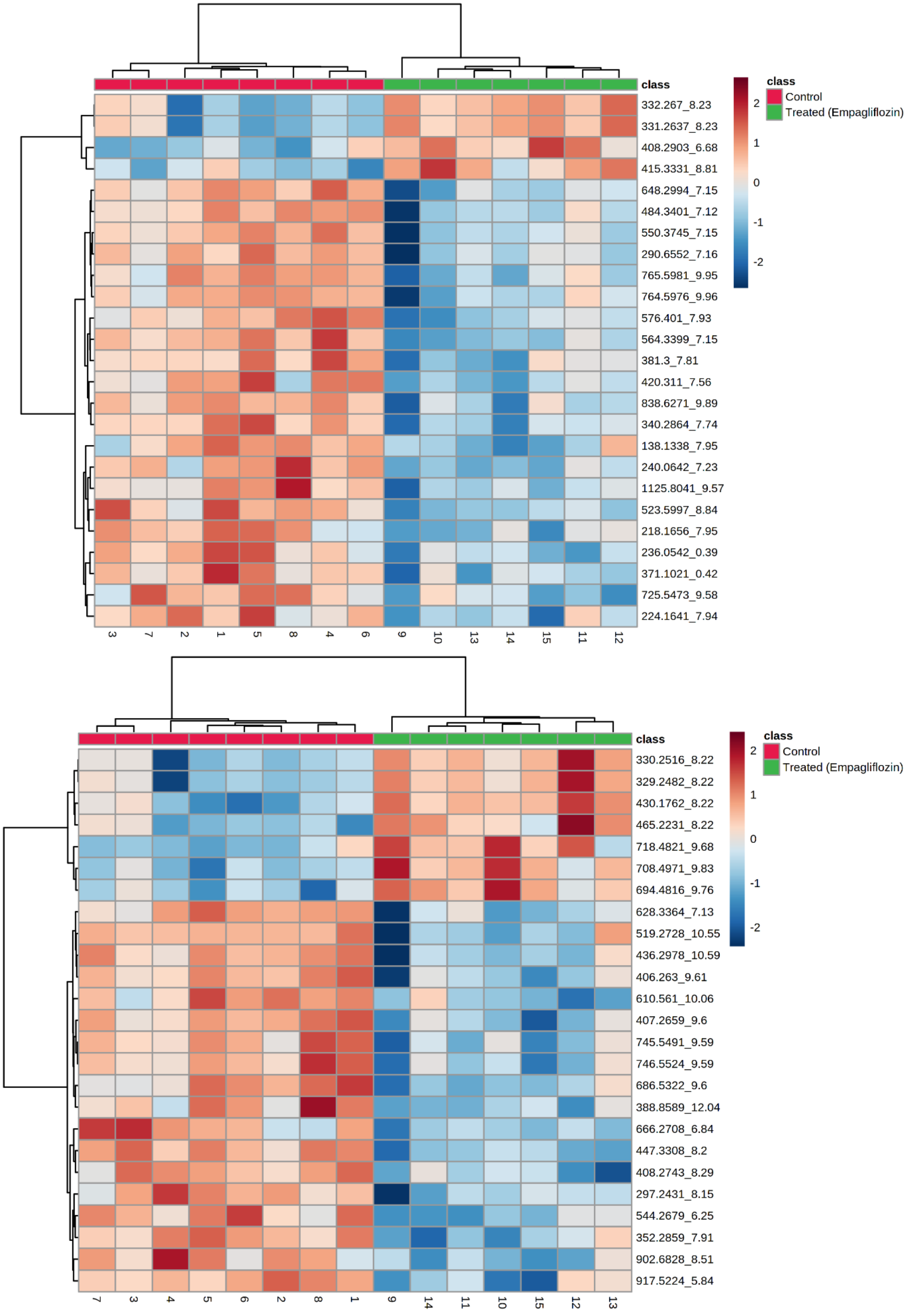

Figure S4- Heatmap of highly changed untargeted metatabolites- Data were normalized, log_{10} transformed and Pareto Scaled prior to one-factor analysis in Metaboanalyst 5.0. Top 25 mz_rt (mass-to-charge ratio and retention time) analytes determined in each mode are shown. A. positive mode; B. negative mode. Distance measure- Euclidean; Cluster method- Ward

Supplemental Table S1- Targeted Metabolites

#	Q1	Q3	Metabolite	Mode
1	282	150.1	1-METHYLADENOSINE_pos_1	Positive
2	170	109.062	1-METHYL-L-HISTIDINE_pos_4	Positive
3	138.1	95	1-METHYLNICOTINAMIDE_pos_1	Positive
4	91	64.9	2,3-BUTANEDIOL_pos_1	Positive
5	104	58	2-AMINOBUTYRIC ACID_pos_1	Positive
6	104.1	57.9	2-AMINOISOBUTYRATE_pos_1	Positive
7	160.1	114.1	2-AMINOOCTANOIC ACID_pos_1	Positive
8	348.1	152.1	2-DEOXYGUANOSINE 5-MONOPHOSPHATE_pos_1	Positive
9	119.1	87	2-HYDROXY-3-METHYLBUTYRIC ACID_pos_4	Positive
10	131	113.2	2-KETOHEXANOIC ACID_pos_2	Positive
11	187.086	98.988	2-PHOSPHOGLYCERATE_pos_4	Positive
12	526	479.8	3,5-DIIODOTHYRONINE_pos_1	Positive
13	104.056	71.967	3-AMINOISOBUTANOIC ACID_pos_1	Positive
14	154	80.1	3-HYDROXYANTHRANILIC ACID_pos_1	Positive
15	105	76.9	3-HYDROXYBUTANOATE_pos_1	Positive
16	119.1	59.924	3-METHYLAMINO-L-ALANINE_pos_2	Positive
17	126.1	95.96	3-METHYLHISTAMINE_pos_2	Positive
18	138	120.1	4-AMINOBENZOIC ACID_pos_1	Positive
19	127.1	80.9	4-IMIDAZOLEACETATE_pos_1	Positive
20	184.01	148.073	4-PYRIDOXATE_pos_1	Positive
21	221.1	204	5-HYDROXYTRYPTOPHAN_pos_1	Positive
22	235.1	176.028	5-METHOXYTRYPTOPHAN_pos_2	Positive
23	460.1	119.031	5-METHYLTETRAHYDROFOLIC ACID_pos_4	Positive
24	323	80.9	5-THYMIDYLIC ACID_pos_1	Positive
25	277	98.987	6-PHOSPHOGLUCONATE_pos_3	Positive
26	298	166	7-METHYLGUANOSINE_pos_1	Positive
27	259.1	109.962	ACADESINE_pos_2	Positive
28	852.1	344.9	ACETOACETYL-COA_pos_1	Positive
29	810.1	303	ACETYL-COA_pos_1	Positive
30	141	123.1	ACETYLPHOSPHATE_pos_1	Positive
31	136	91.9	ADENINE_pos_1	Positive
32	348.048	136.11	ADENOSINE MONOPHOSPHATE_pos_1	Positive
33	508	136.1	ADENOSINE TRIPHOSPHATE_pos_1	Positive
34	268.1	136	ADENOSINE_pos_1	Positive
35	428.018	136.107	ADP_pos_1	Positive
36	590.1	428.1	ADP-GLUCOSE_pos_1	Positive
37	339.1	110	AICAR_pos_1	Positive
38	89.9	44	ALANINE_pos_1	Positive
39	159.04	71.981	ALLANTOIN_pos_2	Positive
40	162	98	AMINOADIPATE_pos_1	Positive
41	138	120	ANTHRANILIC ACID_pos_4	Positive
42	175.1	70	ARGININE_pos_1	Positive
43	291.1	69.9	ARGININOSUCCINIC ACID_pos_1	Positive
44	133.1	86.979	ASPARAGINE_pos_2	Positive
45	134	69.885	ASPARTATE_pos_3	Positive
46	102	58.2	BETAINE ALDEHYDE_pos_1	Positive

47	118.1	58.934	BETAINE_pos_2	Positive
48	245	97	BIOTIN_pos_4	Positive
49	838.2	331	BUTYRYL-COA_pos_1	Positive
50	103.1	86	CADAVERINE_pos_1	Positive
51	227.1	110.1	CARNOSINE_pos_1	Positive
52	404	112	CDP_pos_1	Positive
53	105.1	60.9	CHOLINE_pos_1	Positive
54	132.1	67.917	CIS-4-HYDROXY-D-PROLINE_pos_2	Positive
55	489.079	264.052	CITICOLINE_pos_2	Positive
56	131.1	114.1	CITRACONIC ACID_pos_1	Positive
57	176.1	70	CITRULLINE_pos_2	Positive
58	768.1	261	COENZYME A_pos_1	Positive
59	132.1	90	CREATINE_pos_1	Positive
60	114.1	44	CREATININE_pos_5	Positive
61	678.2	147.1	CYANOCOBALAMIN_+2_pos_3	Positive
62	330.1	136.1	CYCLIC AMP_pos_1	Positive
63	223.044	134.057	CYSTATHIONINE_pos_1	Positive
64	78	60.9	CYSTEAMINE_pos_1	Positive
65	241	152.062	CYSTINE_pos_2	Positive
66	324.033	112.045	CYTIDINE MONOPHOSPHATE_pos_1	Positive
67	244.1	112	CYTIDINE_pos_1	Positive
68	112	68.955	CYTOSINE_pos_1	Positive
69	332.1	136.1	DEOXYADENOSINE MONOPHOSPHATE_pos_1	Positive
70	492	136	DEOXYADENOSINE TRIPHOSPHATE_pos_1	Positive
71	251.993	136	DEOXYADENOSINE_pos_1	Positive
72	308	112	DEOXYCYTIDINE MONOPHOSPHATE_pos_1	Positive
73	268.1	152.1	DEOXYGUANOSINE_pos_1	Positive
74	253.1	109.977	DEOXYINOSINE_pos_2	Positive
75	468.921	80.933	DEOXYURIDINE TRIPHOSPHATE_pos_1	Positive
76	688.2	261	DEPHOSPHO-COA_pos_1	Positive
77	428	152.1	dGDP_pos_1	Positive
78	508.051	152.113	dGTP_pos_1	Positive
79	444.188	297.106	DIHYDROFOLATE_pos_2	Positive
80	171.1	98.9	DIHYDROXYACETONE PHOSPHATE_pos_1	Positive
81	104	58.001	DIMETHYLGLYCINE_pos_1	Positive
82	154.1	137	DOPAMINE_pos_4	Positive
83	483	80.9	dTTP_pos_1	Positive
84	62	44	ETHANOLAMINE_pos_3	Positive
85	786.2	348.1	FAD_pos_1	Positive
86	457.1	438.8	FLAVIN MONONUCLEOTIDE _pos_1	Positive
87	223.1	128.984	FLAVONE_pos_3	Positive
88	442.133	295.115	FOLATE_pos_1	Positive
89	104.054	68.9	GAMMA-AMINOBUTYRATE_pos_1	Positive
90	260.031	144.113	GLUCOSAMINE 6-PHOSPHATE_pos_4	Positive
91	180.04	162	GLUCOSAMINE_pos_6	Positive
92	261	80.844	GLUCOSE 1-PHOSPHATE_pos_2	Positive
93	148	83.9	GLUTAMATE_pos_1	Positive

94	147.1	83.9	GLUTAMINE_pos_1	Positive
95	308.1	179.1	GLUTATHIONE REDUCED_pos_1	Positive
96	258.1	104	GLYCEROPHOSPHOCHOLINE_pos_1	Positive
97	76	76	GLYCINE_pos_2	Positive
98	524	152.1	GTP_pos_pos_2	Positive
99	152	152	GUANINE_pos_3	Positive
100	444.1	152.1	GUANOSINE DIPHOSPHATE_pos_1	Positive
101	364.029	152.1	GUANOSINE MONOPHOSPHATE_pos_1	Positive
102	284	152.1	GUANOSINE_pos_1	Positive
103	180	105.1	HIPPURIC ACID_pos_2	Positive
104	156	110	HISTIDINE_pos_1	Positive
105	142.1	80.9	HISTIDINOL_pos_2	Positive
106	184	138	HOMOCYSTEIC ACID_pos_1	Positive
107	136.013	90.008	HOMOCYSTEINE_pos_2	Positive
108	120	74.1	HOMOSERINE_pos_1	Positive
109	137	110	HYPOXANTHINE_pos_1	Positive
110	429.027	137.119	IDP_pos_2	Positive
111	69	42	IMIDAZOLE_pos_1	Positive
112	118	91.1	INDOLE_pos_1	Positive
113	162.1	118.045	INDOLE-3-CARBOXYLIC ACID_pos_3	Positive
114	188.1	170	INDOLEACRYLIC ACID_pos_1	Positive
115	349.1	137.1	INOSINE MONOPHOSPHATE_pos_1	Positive
116	269.1	137.1	INOSINE_pos_1	Positive
117	132.1	86	ISOLEUCINE_pos_1	Positive
118	209.1	93.976	KYNURENINE_pos_2	Positive
119	204	85	L-ACETYLCARNITINE_pos_1	Positive
120	162.1	103	L-CARNITINE_pos_1	Positive
121	159	79	L-DIHYDROOROTIC ACID_pos_1	Positive
122	132.104	86.002	LEUCINE_pos_1	Positive
123	206	161.2	LIPOAMIDE_pos_1	Positive
124	133.1	69.952	L-ORNITHINE_pos_1	Positive
125	147	90.993	LYSINE_pos_4	Positive
126	854.1	347	MALONYL-COA_pos_1	Positive
127	233.1	174	MELATONIN_pos_1	Positive
128	131	114	MESACONIC ACID_pos_1	Positive
129	198.1	180	METANEPHRINE_pos_1	Positive
130	166.1	73.9	METHIONINE SULFOXIDE_pos_1	Positive
131	150	104	METHIONINE_pos_1	Positive
132	136	118.961	METHYLCYSTEINE_pos_2	Positive
133	298.1	136.1	METHYLTHIOADENOSINE_pos_1	Positive
134	131.1	68.932	MEVALONOLACTONE_pos_2	Positive
135	132.1	85.963	N-ACETYLALANINE_pos_2	Positive
136	222.101	138.1	N-ACETYLGLUCOSAMINE_pos_1	Positive
137	190.1	83.899	N-ACETYLGLUTAMATE_pos_2	Positive
138	189.1	83.903	N-ACETYLGLUTAMINE_pos_2	Positive
139	189.1	172.014	N-ACETYLGLUTAMINE_pos_3	Positive
140	175.1	69.948	N-ACETYLORNITHINE_pos_2	Positive

141	131.1	114	N-ACETYLPUTRESCINE_pos_1	Positive
142	664.2	428.092	NAD_pos_2	Positive
143	666.1	648.8	NADH_pos_1	Positive
144	744.144	604.016	NADP_pos_1	Positive
145	746.1	301.9	NADPH_pos_1	Positive
146	189.1	129.126	N-ALPHA-ACETYLLYSINE_pos_2	Positive
147	173.1	116	N-GLYCYL-L-PROLINE_pos_1	Positive
148	335.052	123.066	NICOTINAMIDE MONONUCLEOTIDE_pos_1	Positive
149	123	80.1	NICOTINAMIDE_pos_1	Positive
150	184.1	134.1	NORMETANEPHRINE_pos_1	Positive
151	148.1	59.941	O-ACETYLSERINE_pos_3	Positive
152	612.9	230.964	OXIDIZED GLUTATHIONE_pos_5	Positive
153	257.1	257.1	PALMITIC ACID_pos_1	Positive
154	220.1	89.9	PANTOTHENATE_pos_1	Positive
155	265	129	PHENYLACETYL-L-GLUTAMINE_pos_5	Positive
156	166.1	120.1	PHENYLALANINE_pos_1	Positive
157	169	80.893	PHOSPHOENOLPYRUVATE_pos_2	Positive
158	184	125	PHOSPHORYLCHOLINE_pos_1	Positive
159	186.029	87.932	PHOSPHOSERINE_pos_3	Positive
160	130.1	67.007	PIPECOLATE_pos_3	Positive
161	116.1	69.9	PROLINE_pos_1	Positive
162	824.1	317	PROPIONYL-COA_pos_1	Positive
163	121	93.9	PURINE_pos_1	Positive
164	89	72	PUTRESCINE_pos_1	Positive
165	169.1	134.1	PYRIDOXAMINE_pos_1	Positive
166	170	134.1	PYRIDOXINE_pos_1	Positive
167	130.046	101.944	PYROGLUTAMIC ACID_pos_2	Positive
168	377.1	243.1	RIBOFLAVIN_pos_1	Positive
169	385.1	136.1	S-ADENOSYLHOMOCYSTEINE_pos_1	Positive
170	399.2	250.1	S-ADENOSYLMETHIONINE_pos_1	Positive
171	90	44	SARCOSINE_pos_1	Positive
172	106.1	59.9	SERINE_pos_2	Positive
173	146.1	112.05	SPERMIDINE_pos_2	Positive
174	146.101	83.932	SPERMIDINE_pos_4	Positive
175	203.1	112.115	SPERMINE_pos_2	Positive
176	868	361	SUCCINYL-COA_pos_1	Positive
177	203.1	69.9	SYMMETRIC DIMETHYLARGININE_pos_1	Positive
178	259.1	241.1	TETRADECANEDIOIC ACID_pos_1	Positive
179	345	122.1	THIAMINE MONOPHOSPHATE_pos_1	Positive
180	425	122	THIAMINE PYROPHOSPHATE_pos_1	Positive
181	266.1	122.085	THIAMINE_pos_2	Positive
182	120.1	74	THREONINE_pos_1	Positive
183	243	127	THYMIDINE_pos_1	Positive
184	127.1	110.03	THYMINE_pos_1	Positive
185	132.091	67.946	TRANS-4-HYDROXY-L-PROLINE_pos_2	Positive
186	205	146.1	TRYPTOPHAN_pos_1	Positive
187	162	143.1	TRYPTOPHANOL_pos_2	Positive

188	182	136.1	TYROSINE_pos_1	Positive
189	113	69.913	URACIL_pos_2	Positive
190	60.9	44	UREA_pos_1	Positive
191	133.1	89.924	UREIDOPROPIONIC ACID_pos_2	Positive
192	177	134.2	UREIDOSUCCINIC ACID_pos_4	Positive
193	405	96.9	URIDINE 5-DIPHOSPHATE_pos_1	Positive
194	485	97	URIDINE TRIPHOSPHATE_pos_1	Positive
195	245	113	URIDINE_pos_1	Positive
196	118.1	54.933	VALINE_pos_2	Positive
197	285.045	153.08	XANTHOSINE_pos_1	Positive
198	206	160	XANTHURENIC ACID_pos_2	Positive
199	365	96.951	XANTHYLIC ACID_pos_2	Positive
200	176	115.4	DBQ_pos	Positive
201	205	171.1	(R)-LIPOIC ACID_neg_1	Negative
202	153	107.9	2,3-DIHYDROXYBENZOIC ACID_neg_1	Negative
203	265	205.233	2,3-DIPHOSPHO-D-GLYCERIC ACID__neg_4	Negative
204	243	96.972	2-DEOXYGLUCOSE-6-PHOSPHATE_neg_4	Negative
205	116.9	117	2-HYDROXY-3-METHYLBUTYRIC ACID_neg_1	Negative
206	103	103	2-HYDROXYBUTYRATE_neg_3	Negative
207	174.9	115.05	2-ISOPROPYLMALIC ACID_neg_2	Negative
208	129.001	129	2-KETOHEXANOIC ACID_neg_1	Negative
209	179	107	3-(4-HYDROXYPHENYL)PYRUVATE_neg_1	Negative
210	454.7	78.8	3-HYDROXY-3-METHYLGLUTARYL-COA_+2_neg_3	Negative
211	425.8	382.8	3-HYDROXYBUTYRYL-CoA_-2_neg_4	Negative
212	151	107	3-HYDROXYPHENYLACETIC ACID_neg_1	Negative
213	129	129	3-METHYL-2-OXOVALERIC ACID_neg_1	Negative
214	136.001	92	4-AMINOBENZOIC ACID_neg_1	Negative
215	137	92.8	4-HYDROXYBENZOATE_neg_1	Negative
216	181.937	108.07	4-PYRIDOXATE_neg_2	Negative
217	219	132.102	5-HYDROXYTRYPTOPHAN_neg_2	Negative
218	320.9	195.068	5-THYMIDYLIC ACID_neg_1	Negative
219	274.843	96.921	6-PHOSPHOGLUCONATE_neg_2	Negative
220	257	125.096	ACADESINE_neg_2	Negative
221	101	56.9	ACETOACETATE_neg_2	Negative
222	850.138	765.84	ACETOACETYL-COA_neg_1	Negative
223	808.127	407.958	ACETYL-COA_neg_1	Negative
224	345.941	134.09	ADENOSINE MONOPHOSPHATE_neg_2	Negative
225	426.016	346.024	ADENOSINE PHOSPHOSULFATE_neg_2	Negative
226	505.8	158.969	ADENOSINE TRIPHOSPHATE_neg_2	Negative
227	425.831	134.11	ADP_neg_2	Negative
228	587.958	346.107	ADP-GLUCOSE_neg_1	Negative
229	336.9	78.8	AICAR_neg_1	Negative
230	156.927	114.077	ALLANTOIN_neg_2	Negative
231	179	89	ALPHA-D-GLUCOSE_neg_3	Negative
232	288.9	131.12	ARGININOSUCCINIC ACID_neg_2	Negative
233	174.887	115.066	ASCORBATE_neg_2	Negative
234	164.9	121.091	ATROLACTIC ACID_neg_2	Negative

235	243	41.9	BIOTIN_neg_2	Negative
236	836.159	835.861	BUTYRYL-COA_neg_2	Negative
237	139.9	79	CARBAMOYL PHOSPHATE_neg_1	Negative
238	401.9	158.947	CDP_neg_2	Negative
239	465.2	96.934	CHOLESTERYL SULFATE_neg_2	Negative
240	129.1	85.1	CITRACONIC ACID_neg	Negative
241	146.9	87	CITRAMALATE_neg_1	Negative
242	146.9	85.041	CITRAMALATE_neg_2	Negative
243	191	111	CITRIC ACID_neg_1	Negative
244	327.898	133.994	CYCLIC AMP_neg_1	Negative
245	220.955	134.091	CYSTATHIONINE_neg_1	Negative
246	119.932	119.932	CYSTEINE_neg_5	Negative
247	321.908	211.057	CYTIDINE MONOPHOSPHATE_neg_1	Negative
248	466	158.9	dCTP_neg_1	Negative
249	489.9	158.951	DEOXYADENOSINE TRIPHOSPHATE_neg_2	Negative
250	266	150.1	DEOXYGUANOSINE_neg_1	Negative
251	251	135.08	DEOXYINOSINE_neg_2	Negative
252	213	96.974	DEOXYRIBOSE 5-PHOSPHATE_neg_2	Negative
253	226.997	184.193	DEOXYURIDINE_neg_1	Negative
254	686	339.125	DEPHOSPHO-COA_neg_2	Negative
255	199	96.8	D-Erythrose 4-phosphate_neg_2	Negative
256	425.866	158.965	dGDP_neg_2	Negative
257	506	78.9	dGTP_neg_3	Negative
258	442.003	133.094	DIHYDROFOLATE_neg_3	Negative
259	168.906	78.842	DIHYDROXYACETONE PHOSPHATE_neg_2	Negative
260	229	96.942	D-RIBOSE 5-PHOSPHATE_neg_2	Negative
261	288.9	289	D-SEDOHEPTULOSE 7-PHOSPHATE_neg_1	Negative
262	321.2	195	dTDP_-HPO3_neg_4	Negative
263	401	274.8	dTDP_neg_2	Negative
264	480.9	480.8	dTTP_neg_1	Negative
265	784	437.194	FAD_neg_1	Negative
266	381.3	158.8	FARNESYL PYROPHOSPHATE_neg_2	Negative
267	455.102	213	FLAVIN MONONUCLEOTIDE _neg_1	Negative
268	339	96.922	FRUCTOSE 1,6-BISPHOSPHATE_neg_2	Negative
269	258.9	78.856	FRUCTOSE 6-PHOSPHATE_neg_2	Negative
270	115	27	FUMARIC ACID_neg_1	Negative
271	233.3	96.8	GERANYL-PP_-HPO3_neg_2	Negative
272	313	78.7	GERANYL-PP_neg_1	Negative
273	194.922	74.881	GLUCONATE_neg_2	Negative
274	176.788	129.167	GLUCONOLACTONE_neg_4	Negative
275	257.969	96.922	GLUCOSAMINE 6-PHOSPHATE_neg_2	Negative
276	258.9	79	GLUCOSE 1-PHOSPHATE_neg_1	Negative
277	258.879	78.814	GLUCOSE 6-PHOSPHATE_neg_1	Negative
278	192.943	72.897	GLUCURONATE_neg_3	Negative
279	305.9	143.1	GLUTATHIONE REDUCED_neg_1	Negative
280	169	58.9	GLYCERALDEHYDE 3-PHOSPHATE_neg_1	Negative
281	104.912	74.868	GLYCERATE_neg_1	Negative

282	170.9	79	GLYCEROL 3-PHOSPHATE_neg_1	Negative
283	75	44.8	GLYCOLATE_neg_2	Negative
284	73	44.9	GLYOXYLIC ACID_neg_1	Negative
285	522	159.1	GTP_neg_1	Negative
286	441.9	78.8	GUANOSINE DIPHOSPHATE_neg_1	Negative
287	361.9	78.8	GUANOSINE MONOPHOSPHATE_neg_1	Negative
288	178	134	HIPPURIC ACID_neg_2	Negative
289	181.9	80	HOMOCYSTEIC ACID_neg_1	Negative
290	118	72	HOMOSERINE_neg_2	Negative
291	131	85.063	HYDROXYISOCAPROIC ACID_neg_2	Negative
292	426.842	329.093	IDP_neg_4	Negative
293	116	40.1	INDOLE_neg_2	Negative
294	160	116.1	INDOLE-3-CARBOXYLIC ACID_neg_1	Negative
295	186	142.096	INDOLEACRYLIC ACID_neg_2	Negative
296	144	73.8	ISOBUTYRYLGLYCINE_neg_2	Negative
297	190.893	72.924	ISOCITRATE_neg_4	Negative
298	101	101	ISOVALERIC ACID_neg_1	Negative
299	146.9	129	L-2-HYDROXYGLUTARIC ACID_neg_1	Negative
300	89	42.9	LACTATE_neg_1	Negative
301	157	41.9	L-DIHYDROOROTIC ACID_neg_1	Negative
302	130.9	83.04	L-ORNITHINE_neg_4	Negative
303	115	27.1	MALEIC ACID_neg_2	Negative
304	132.898	115	MALIC ACID_neg_5	Negative
305	852.117	407.987	MALONYL-COA_neg_2	Negative
306	231	144.035	MELATONIN_neg_2	Negative
307	129	85	MESACONIC ACID_neg_1	Negative
308	149	105	METHYL PHENYLACETATE_neg_3	Negative
309	117	72.8	METHYLMALONATE_neg_1	Negative
310	131	87.05	METHYLSUCCINIC ACID_neg_2	Negative
311	147	40.9	MEVALONIC ACID_neg_2	Negative
312	179	161	MYOINOSITOL_neg_3	Negative
313	129.9	87.9	N-ACETYLALANINE_neg_1	Negative
314	299.9	78.98	N-ACETYL-GLUCOSAMINE 1-PHOSPHATE_neg_2	Negative
315	188	128.1	N-ACETYLGLUTAMATE_neg_1	Negative
316	187	127.1	N-ACETYLGLUTAMINE_neg_1	Negative
317	172.9	131.146	N-ACETYLORNITHINE_neg_3	Negative
318	741.974	619.963	NADP_neg_1	Negative
319	744	408	NADPH_neg_1	Negative
320	171	114.117	N-GLYCYL-L-PROLINE_neg_3	Negative
321	145.934	116.028	O-ACETYLSERINE_neg_2	Negative
322	154.896	110.993	OROTATE_neg_1	Negative
323	131	86.9	OXALACETIC ACID_neg_1	Negative
324	611	306	OXIDIZED GLUTATHIONE_neg_1	Negative
325	144.9	56.9	OXOGLUTARATE_neg_2	Negative
326	255	255	PALMITIC ACID_neg_1	Negative
327	218	88	PANTOTHENATE_neg_1	Negative
328	263	263	PHENYLACETYL-L-GLUTAMINE_neg_2	Negative

329	165	103.129	PHENYLLACTIC ACID_neg_3	Negative
330	144.9	101.1	PHENYLPROPIOLIC ACID_neg_1	Negative
331	163	90.8	PHENYLPYRUVATE_neg_2	Negative
332	166.826	78.855	PHOSPHOENOLPYRUVATE_neg_1	Negative
333	388.949	291	PHOSPHORIBOSYL PYROPHOSPHATE_neg_1	Negative
334	822.143	407.951	PROPIONYL-COA_neg_1	Negative
335	127.932	83.969	PYROGLUTAMIC ACID_neg_2	Negative
336	176.9	115.007	PYROPHOSPHATE_neg_3	Negative
337	87	42.8	PYRUVIC ACID_neg_1	Negative
338	375	255.1	RIBOFLAVIN_neg_1	Negative
339	229.015	78.988	RIBULOSE 5-PHOSPHATE_neg_2	Negative
340	383	134	S-ADENOSYLHOMOCYSTEINE_neg_1	Negative
341	116.903	72.92	SUCCINATE_neg_1	Negative
342	200.9	131.124	SYMMETRIC DIMETHYLARGININE_neg_2	Negative
343	128	80	Taurine_D4_neg	Negative
344	123.906	79.839	TAURINE_neg_1	Negative
345	257.1	239.216	TETRADECANEDIOIC ACID_neg_2	Negative
346	423	301.879	THIAMINE PYROPHOSPHATE_neg_2	Negative
347	241	42	THYMIDINE_neg_2	Negative
348	172.859	84.934	TRANS-ACONITATE_neg_1	Negative
349	203	115.9	TRYPTOPHAN_neg_1	Negative
350	130.9	131	UREIDOPROPIONIC ACID_neg_1	Negative
351	175	87.9	UREIDOSUCCINIC ACID_neg_2	Negative
352	402.9	158.937	URIDINE 5-DIPHOSPHATE_neg_2	Negative
353	564.9	323.048	URIDINE DIPHOSPHATE GLUCOSE_neg_2	Negative
354	482.9	78.8	URIDINE TRIPHOSPHATE_neg_1	Negative
355	243.001	41.9	URIDINE_neg_2	Negative
356	282.97	151.066	XANTHOSINE_neg_1	Negative
357	204	115.9	XANTHURENIC ACID_neg_1	Negative
358	362.9	211.037	XANTHYLIC ACID_neg_2	Negative
359	166	122	4-Nitrobenzoic acid_neg_1	Negative
360	166	92	4-Nitrobenzoic acid_neg_2	Negative

Supplemental Table S2- Targeted Lipids

	Q1	Q3	Metabolite	Mode
1	675.6	369.4	IS_CE	Positive
2	586.6	369.4	CE(12:0)	Positive
3	614.6	369.4	CE(14:0)	Positive
4	612.6	369.4	CE(14:1)	Positive
5	628.6	369.4	CE(15:0)	Positive
6	642.6	369.4	CE(16:0)	Positive
7	640.6	369.4	CE(16:1)	Positive
8	656.6	369.4	CE(17:0)	Positive
9	670.6	369.4	CE(18:0)	Positive
10	668.6	369.4	CE(18:1)	Positive
11	666.6	369.4	CE(18:2)	Positive
12	664.6	369.4	CE(18:3)	Positive
13	662.6	369.4	CE(18:4)	Positive
14	698.7	369.4	CE(20:0)	Positive
15	696.7	369.4	CE(20:1)	Positive
16	694.6	369.4	CE(20:2)	Positive
17	692.6	369.4	CE(20:3)	Positive
18	690.6	369.4	CE(20:4)	Positive
19	688.6	369.4	CE(20:5)	Positive
20	726.7	369.4	CE(22:0)	Positive
21	724.7	369.4	CE(22:1)	Positive
22	722.7	369.4	CE(22:2)	Positive
23	718.6	369.4	CE(22:4)	Positive
24	716.6	369.4	CE(22:5)	Positive
25	714.6	369.4	CE(22:6)	Positive
26	754.7	369.4	CE(24:0)	Positive
27	752.7	369.4	CE(24:1)	Positive
28	547.6	264.4	IS_CER_2	Positive
29	531.8	294.9	IS_CER(d7_15:0)	Positive
30	531.8	264.4	IS_CER(d7_15:0)_2	Positive
31	482.6	264.4	IS_CER_1	Positive
32	510.6	264.4	CER(14:0)	Positive
33	538.6	264.4	CER(16:0)	Positive
34	566.7	264.4	CER(18:0)	Positive
35	564.8	264.4	CER(18:1)	Positive
36	594.6	264.4	CER(20:0)	Positive
37	592.6	264.4	CER(20:1)	Positive
38	622.7	264.4	CER(22:0)	Positive
39	620.7	264.4	CER(22:1)	Positive
40	650.8	264.4	CER(24:0)	Positive
41	648.8	264.4	CER(24:1)	Positive
42	678.9	264.4	CER(26:0)	Positive
43	676.9	264.4	CER(26:1)	Positive
44	393.4	393.4	Cholesterol (d7)	Positive
45	369.3	369.3	Cholesterol	Positive
46	605.58	346.3	IS_DAG	Positive

47	530.4	257.2	DAG(12:0/16:0)	Positive	16:0)
48	558.5	257.2	DAG(12:0/18:0)	Positive	18:0)
49	556.5	257.2	DAG(12:0/18:1)	Positive	18:1)
50	554.4	257.2	DAG(12:0/18:2)	Positive	18:2)
51	530.4	285.2	DAG(14:0/14:0)	Positive	14:0)
52	558.5	285.2	DAG(14:0/16:0)	Positive	16:0)
53	556.5	285.2	DAG(14:0/16:1)	Positive	16:1)
54	584.4	285.2	DAG(14:0/18:1)	Positive	18:1)
55	582.4	285.2	DAG(14:0/18:2)	Positive	18:2)
56	580.4	285.2	DAG(14:0/18:3)	Positive	18:3)
57	614.6	285.2	DAG(14:0/20:0)	Positive	20:0)
58	606.4	285.2	DAG(14:0/20:4)	Positive	20:4)
59	630.5	285.3	DAG(14:0/22:6)	Positive	22:6)
60	556.5	283.2	DAG(14:1/16:0)	Positive	16:0)
61	582.4	283.2	DAG(14:1/18:1)	Positive	18:1)
62	598.5	299.3	DAG(15:0/18:1)	Positive	18:1)
63	596.5	299.3	DAG(15:0/18:2)	Positive	18:2)
64	586.5	313.3	DAG(16:0/16:0)	Positive	16:0)
65	584.4	313.2	DAG(16:0/16:1)	Positive	16:1)
66	614.4	313.2	DAG(16:0/18:0)	Positive	18:0)
67	612.6	313.2	DAG(16:0/18:1)	Positive	18:1)
68	610.4	313.2	DAG(16:0/18:2)	Positive	18:2)
69	608.5	313.2	DAG(16:0/18:3)	Positive	18:3)
70	636.5	313.3	DAG(16:0/20:3)	Positive	20:3)
71	634.5	313.3	DAG(16:0/20:4)	Positive	20:4)
72	632.4	313.3	DAG(16:0/20:5)	Positive	20:5)
73	660.5	313.3	DAG(16:0/22:5)	Positive	22:5)
74	658.5	313.3	DAG(16:0/22:6)	Positive	22:6)
75	582.4	311.2	DAG(16:1/16:1)	Positive	16:1)
76	612.6	311.3	DAG(16:1/18:0)	Positive	18:0)
77	610.4	311.2	DAG(16:1/18:1)	Positive	18:1)
78	608.5	311.2	DAG(16:1/18:2)	Positive	18:2)
79	606.4	311.2	DAG(16:1/18:3)	Positive	18:3)
80	636.6	311.3	DAG(16:1/20:2)	Positive	20:2)
81	632.4	311.3	DAG(16:1/20:4)	Positive	20:4)
82	656.5	311.3	DAG(16:1/22:6)	Positive	22:6)
83	640.4	341.3	DAG(18:0/18:1)	Positive	18:1)
84	638.4	341.3	DAG(18:0/18:2)	Positive	18:2)
85	636.5	341.3	DAG(18:0/18:3)	Positive	18:3)
86	686.6	341.3	DAG(18:0/22:6)	Positive	22:6)
87	638.4	339.3	DAG(18:1/18:1)	Positive	18:1)
88	636.5	339.3	DAG(18:1/18:2)	Positive	18:2)
89	666.6	339.3	DAG(18:1/20:1)	Positive	20:1)
90	664.6	339.3	DAG(18:1/20:2)	Positive	20:2)
91	662.6	339.3	DAG(18:1/20:3)	Positive	20:3)
92	660.5	339.3	DAG(18:1/20:4)	Positive	20:4)
93	658.5	339.3	DAG(18:1/20:5)	Positive	20:5)

94	688.6	339.3	DAG(18:1/22:4)	Positive	22:4)
95	686.6	339.3	DAG(18:1/22:5)	Positive	22:5)
96	684.6	339.3	DAG(18:1/22:6)	Positive	22:6)
97	632.4	337.3	DAG(18:2/18:3)	Positive	18:3)
98	660.5	337.3	DAG(18:2/20:3)	Positive	20:3)
99	658.5	337.3	DAG(18:2/20:4)	Positive	20:4)
100	656.5	337.3	DAG(18:2/20:5)	Positive	20:5)
101	686.6	337.3	DAG(18:2/22:4)	Positive	22:4)
102	684.6	337.3	DAG(18:2/22:5)	Positive	22:5)
103	682.5	337.3	DAG(18:2/22:6)	Positive	22:6)
104	698.6	369.3	DAG(20:0/20:0)	Positive	20:0)
105	549.6	266.4	IS_DCER (16:0)	Positive	
106	540.6	266.4	DCER(16:0)	Positive	
107	568.7	266.4	DCER(18:0)	Positive	
108	596.7	266.4	DCER(20:0)	Positive	
109	594.4	266.4	DCER(20:1)	Positive	
110	624.8	266.4	DCER(22:0)	Positive	
111	620.4	266.4	DCER(22:1)	Positive	
112	652.9	266.4	DCER(24:0)	Positive	
113	650.9	266.4	DCER(24:1)	Positive	
114	680.5	266.4	DCER(26:0)	Positive	
115	709.7	264.4	IS_HCER (16:0)	Positive	
116	672.5	264.4	HCER(14:0)	Positive	
117	700.7	264.4	HCER(16:0)	Positive	
118	728.8	264.4	HCER(18:0)	Positive	
119	726.7	264.4	HCER(18:1)	Positive	
120	756.7	264.4	HCER(20:0)	Positive	
121	754.7	264.4	HCER(20:1)	Positive	
122	784.9	264.4	HCER(22:0)	Positive	
123	782.8	264.4	HCER(22:1)	Positive	
124	812.9	264.4	HCER(24:0)	Positive	
125	810.9	264.4	HCER(24:1)	Positive	
126	840.9	264.4	HCER(26:0)	Positive	
127	838.9	264.4	HCER(26:1)	Positive	
128	871.9	264.4	dLCER(16:0)	Positive	
129	834.9	264.4	LCER(14:0)	Positive	
130	862.9	264.4	LCER(16:0)	Positive	
131	890.2	264.4	LCER(18:0)	Positive	
132	888.2	264.4	LCER(18:1)	Positive	
133	918.2	264.4	LCER(20:0)	Positive	
134	916.2	264.4	LCER(20:1)	Positive	
135	946.2	264.4	LCER(22:0)	Positive	
136	944.2	264.4	LCER(22:1)	Positive	
137	974.8	264.4	LCER(24:0)	Positive	
138	972.9	264.4	LCER(24:1)	Positive	
139	364.4	364.4	IS_MAG_d7_(18:1)	Positive	
140	331.3	313.3	MAG(16:0)	Positive	

141	329.3	311.3	MAG(16:1)	Positive	
142	359.3	341.3	MAG(18:0)	Positive	
143	357.3	339.3	MAG(18:1)	Positive	
144	355.3	337.3	MAG(18:2)	Positive	
145	381.3	363.3	MAG(20:3)	Positive	
146	379.3	361.3	MAG(20:4)	Positive	
147	405.3	387.3	MAG(22:5)	Positive	
148	742.5	335.3	PE(14:0/22:2)	Positive	22:2)
149	738.5	331.3	PE(14:0/22:4)	Positive	22:4)
150	736.5	329.2	PE(14:0/22:5)	Positive	22:5)
151	734.5	327.2	PE(14:0/22:6)	Positive	22:6)
152	646.4	241.2	PE(15:0/14:1)	Positive	14:1)
153	660.5	225.2	PE(16:0/14:1)	Positive	14:1)
154	686.5	281.2	PE(18:1/14:1)	Positive	14:1)
155	684.5	279.2	PE(18:2/14:1)	Positive	14:1)
156	738.6	184.1	IS_SM	Positive	
157	647.5	184.1	SM(d18:1/12:0)	Positive	12:0)
158	675.5	184.1	SM(d18:1/14:0)	Positive	14:0)
159	673.5	184.1	SM(d18:1/14:1)	Positive	14:1)
160	703.6	184.1	SM(d18:1/16:0)	Positive	16:0)
161	701.6	184.1	SM(d18:1/16:1)	Positive	16:1)
162	717.6	184.1	SM(d18:1/17:0)	Positive	17:0)
163	731.6	184.1	SM(d18:1/18:0)	Positive	18:0)
164	729.6	184.1	SM(d18:1/18:1)	Positive	18:1)
165	727.6	184.1	SM(d18:1/18:2)	Positive	18:2)
166	725.6	184.1	SM(d18:1/18:3)	Positive	18:3)
167	723.5	184.1	SM(d18:1/18:4)	Positive	18:4)
168	759.6	184.1	SM(d18:1/20:0)	Positive	20:0)
169	757.6	184.1	SM(d18:1/20:1)	Positive	20:1)
170	755.6	184.1	SM(d18:1/20:2)	Positive	20:2)
171	753.6	184.1	SM(d18:1/20:3)	Positive	20:3)
172	751.6	184.1	SM(d18:1/20:4)	Positive	20:4)
173	749.6	184.1	SM(d18:1/20:5)	Positive	20:5)
174	787.7	184.1	SM(d18:1/22:0)	Positive	22:0)
175	785.7	184.1	SM(d18:1/22:1)	Positive	22:1)
176	783.6	184.1	SM(d18:1/22:2)	Positive	22:2)
177	781.6	184.1	SM(d18:1/22:3)	Positive	22:3)
178	779.6	184.1	SM(d18:1/22:4)	Positive	22:4)
179	777.6	184.1	SM(d18:1/22:5)	Positive	22:5)
180	775.6	184.1	SM(d18:1/22:6)	Positive	22:6)
181	815.7	184.1	SM(24:0)	Positive	
182	813.7	184.1	SM(24:1)	Positive	
183	843.7	184.1	SM(26:0)	Positive	
184	841.7	184.1	SM(26:1)	Positive	
185	829.8	570.56	IS_TAG	Positive	
186	824.7	579.5	TAG48:0-FA14:0	Positive	
187	824.7	551.4	TAG48:0-FA16:0	Positive	

188	824.7	523.4	TAG48:0-FA18:0	Positive
189	822.7	605.5	TAG48:1-FA12:0	Positive
190	822.7	577.5	TAG48:1-FA14:0	Positive
191	822.7	579.5	TAG48:1-FA14:1	Positive
192	822.7	549.4	TAG48:1-FA16:0	Positive
193	822.7	551.4	TAG48:1-FA16:1	Positive
194	822.7	521.4	TAG48:1-FA18:0	Positive
195	822.7	523.4	TAG48:1-FA18:1	Positive
196	820.7	603.5	TAG48:2-FA12:0	Positive
197	820.7	575.5	TAG48:2-FA14:0	Positive
198	820.7	577.5	TAG48:2-FA14:1	Positive
199	820.7	547.4	TAG48:2-FA16:0	Positive
200	820.7	549.4	TAG48:2-FA16:1	Positive
201	820.7	519.4	TAG48:2-FA18:0	Positive
202	820.7	521.4	TAG48:2-FA18:1	Positive
203	820.7	523.4	TAG48:2-FA18:2	Positive
204	818.7	601.5	TAG48:3-FA12:0	Positive
205	818.7	573.5	TAG48:3-FA14:0	Positive
206	818.7	575.5	TAG48:3-FA14:1	Positive
207	818.7	545.4	TAG48:3-FA16:0	Positive
208	818.7	547.4	TAG48:3-FA16:1	Positive
209	818.7	519.4	TAG48:3-FA18:1	Positive
210	818.7	521.4	TAG48:3-FA18:2	Positive
211	818.7	523.4	TAG48:3-FA18:3	Positive
212	816.7	599.5	TAG48:4-FA12:0	Positive
213	816.7	571.5	TAG48:4-FA14:0	Positive
214	816.7	573.5	TAG48:4-FA14:1	Positive
215	816.7	543.4	TAG48:4-FA16:0	Positive
216	816.7	545.4	TAG48:4-FA16:1	Positive
217	816.7	517.4	TAG48:4-FA18:1	Positive
218	816.7	519.4	TAG48:4-FA18:2	Positive
219	816.7	521.4	TAG48:4-FA18:3	Positive
220	816.7	495.4	TAG48:4-FA20:4	Positive
221	814.7	517.4	TAG48:5-FA18:2	Positive
222	814.7	519.4	TAG48:5-FA18:3	Positive
223	852.8	607.6	TAG50:0-FA14:0	Positive
224	852.8	579.5	TAG50:0-FA16:0	Positive
225	852.8	551.5	TAG50:0-FA18:0	Positive
226	850.8	605.6	TAG50:1-FA14:0	Positive
227	850.8	577.5	TAG50:1-FA16:0	Positive
228	850.8	579.5	TAG50:1-FA16:1	Positive
229	850.8	549.5	TAG50:1-FA18:0	Positive
230	850.8	551.5	TAG50:1-FA18:1	Positive
231	850.8	523.5	TAG50:1-FA20:1	Positive
232	848.8	603.6	TAG50:2-FA14:0	Positive
233	848.8	605.6	TAG50:2-FA14:1	Positive
234	848.8	575.5	TAG50:2-FA16:0	Positive

235	848.8	577.5	TAG50:2-FA16:1	Positive
236	848.8	547.5	TAG50:2-FA18:0	Positive
237	848.8	549.5	TAG50:2-FA18:1	Positive
238	848.8	551.5	TAG50:2-FA18:2	Positive
239	848.8	523.5	TAG50:2-FA20:2	Positive
240	846.8	601.6	TAG50:3-FA14:0	Positive
241	846.8	603.6	TAG50:3-FA14:1	Positive
242	846.8	573.5	TAG50:3-FA16:0	Positive
243	846.8	575.5	TAG50:3-FA16:1	Positive
244	846.8	545.5	TAG50:3-FA18:0	Positive
245	846.8	547.5	TAG50:3-FA18:1	Positive
246	846.8	549.5	TAG50:3-FA18:2	Positive
247	846.8	551.5	TAG50:3-FA18:3	Positive
248	846.8	523.5	TAG50:3-FA20:3	Positive
249	844.6	599.4	TAG50:4-FA14:0	Positive
250	844.6	601.4	TAG50:4-FA14:1	Positive
251	844.6	571.3	TAG50:4-FA16:0	Positive
252	844.6	573.3	TAG50:4-FA16:1	Positive
253	844.6	545.3	TAG50:4-FA18:1	Positive
254	844.6	547.3	TAG50:4-FA18:2	Positive
255	844.6	549.3	TAG50:4-FA18:3	Positive
256	844.6	521.3	TAG50:4-FA20:3	Positive
257	844.6	523.3	TAG50:4-FA20:4	Positive
258	842.6	597.4	TAG50:5-FA14:0	Positive
259	842.6	569.3	TAG50:5-FA16:0	Positive
260	842.6	571.3	TAG50:5-FA16:1	Positive
261	842.6	543.3	TAG50:5-FA18:1	Positive
262	842.6	545.3	TAG50:5-FA18:2	Positive
263	842.6	547.3	TAG50:5-FA18:3	Positive
264	842.6	521.3	TAG50:5-FA20:4	Positive
265	842.6	523.3	TAG50:5-FA20:5	Positive
266	840.7	519.4	TAG50:6-FA20:4	Positive
267	880.8	607.5	TAG52:0-FA16:0	Positive
268	880.8	579.5	TAG52:0-FA18:0	Positive
269	880.8	551.5	TAG52:0-FA20:0	Positive
270	878.8	605.5	TAG52:1-FA16:0	Positive
271	878.8	607.5	TAG52:1-FA16:1	Positive
272	878.8	577.5	TAG52:1-FA18:0	Positive
273	878.8	579.5	TAG52:1-FA18:1	Positive
274	878.8	549.5	TAG52:1-FA20:0	Positive
275	878.8	551.5	TAG52:1-FA20:1	Positive
276	876.8	631.6	TAG52:2-FA14:0	Positive
277	876.8	603.5	TAG52:2-FA16:0	Positive
278	876.8	605.5	TAG52:2-FA16:1	Positive
279	876.8	575.5	TAG52:2-FA18:0	Positive
280	876.8	577.5	TAG52:2-FA18:1	Positive
281	876.8	579.5	TAG52:2-FA18:2	Positive

282	876.8	547.5	TAG52:2-FA20:0	Positive
283	876.8	549.5	TAG52:2-FA20:1	Positive
284	876.8	551.5	TAG52:2-FA20:2	Positive
285	874.8	629.6	TAG52:3-FA14:0	Positive
286	874.8	601.5	TAG52:3-FA16:0	Positive
287	874.8	603.5	TAG52:3-FA16:1	Positive
288	874.8	573.5	TAG52:3-FA18:0	Positive
289	874.8	575.5	TAG52:3-FA18:1	Positive
290	874.8	577.5	TAG52:3-FA18:2	Positive
291	874.8	579.5	TAG52:3-FA18:3	Positive
292	874.8	545.5	TAG52:3-FA20:0	Positive
293	874.8	547.5	TAG52:3-FA20:1	Positive
294	874.8	549.5	TAG52:3-FA20:2	Positive
295	874.8	551.5	TAG52:3-FA20:3	Positive
296	874.8	519.5	TAG52:3-FA22:1	Positive
297	872.8	627.6	TAG52:4-FA14:0	Positive
298	872.8	599.5	TAG52:4-FA16:0	Positive
299	872.8	601.5	TAG52:4-FA16:1	Positive
300	872.8	571.5	TAG52:4-FA18:0	Positive
301	872.8	573.5	TAG52:4-FA18:1	Positive
302	872.8	575.5	TAG52:4-FA18:2	Positive
303	872.8	577.5	TAG52:4-FA18:3	Positive
304	872.8	543.5	TAG52:4-FA20:0	Positive
305	872.8	547.5	TAG52:4-FA20:2	Positive
306	872.8	549.5	TAG52:4-FA20:3	Positive
307	872.8	551.5	TAG52:4-FA20:4	Positive
308	872.8	517.5	TAG52:4-FA22:1	Positive
309	872.8	523.5	TAG52:4-FA22:4	Positive
310	870.8	625.6	TAG52:5-FA14:0	Positive
311	870.8	597.5	TAG52:5-FA16:0	Positive
312	870.8	599.5	TAG52:5-FA16:1	Positive
313	870.8	571.5	TAG52:5-FA18:1	Positive
314	870.8	573.5	TAG52:5-FA18:2	Positive
315	870.8	575.5	TAG52:5-FA18:3	Positive
316	870.8	547.5	TAG52:5-FA20:3	Positive
317	870.8	549.5	TAG52:5-FA20:4	Positive
318	870.8	551.5	TAG52:5-FA20:5	Positive
319	870.8	523.5	TAG52:5-FA22:5	Positive
320	868.8	623.6	TAG52:6-FA14:0	Positive
321	868.8	595.5	TAG52:6-FA16:0	Positive
322	868.8	597.5	TAG52:6-FA16:1	Positive
323	868.8	569.5	TAG52:6-FA18:1	Positive
324	868.8	571.5	TAG52:6-FA18:2	Positive
325	868.8	573.5	TAG52:6-FA18:3	Positive
326	868.8	547.5	TAG52:6-FA20:4	Positive
327	868.8	549.5	TAG52:6-FA20:5	Positive
328	868.8	523.5	TAG52:6-FA22:6	Positive

329	866.7	593.4	TAG52:7-FA16:0	Positive
330	866.7	567.4	TAG52:7-FA18:1	Positive
331	866.7	547.4	TAG52:7-FA20:5	Positive
332	866.7	521.4	TAG52:7-FA22:6	Positive
333	864.8	593.5	TAG52:8-FA16:1	Positive
334	864.8	567.5	TAG52:8-FA18:2	Positive
335	908.8	635.5	TAG54:0-FA16:0	Positive
336	908.8	607.5	TAG54:0-FA18:0	Positive
337	906.8	633.5	TAG54:1-FA16:0	Positive
338	906.8	605.5	TAG54:1-FA18:0	Positive
339	906.8	607.5	TAG54:1-FA18:1	Positive
340	906.8	577.5	TAG54:1-FA20:0	Positive
341	906.8	579.5	TAG54:1-FA20:1	Positive
342	904.8	631.5	TAG54:2-FA16:0	Positive
343	904.8	603.5	TAG54:2-FA18:0	Positive
344	904.8	605.5	TAG54:2-FA18:1	Positive
345	904.8	607.5	TAG54:2-FA18:2	Positive
346	904.8	575.5	TAG54:2-FA20:0	Positive
347	904.8	577.5	TAG54:2-FA20:1	Positive
348	904.8	579.5	TAG54:2-FA20:2	Positive
349	902.8	629.5	TAG54:3-FA16:0	Positive
350	902.8	631.5	TAG54:3-FA16:1	Positive
351	902.8	601.5	TAG54:3-FA18:0	Positive
352	902.8	603.5	TAG54:3-FA18:1	Positive
353	902.8	605.5	TAG54:3-FA18:2	Positive
354	902.8	607.5	TAG54:3-FA18:3	Positive
355	902.8	575.5	TAG54:3-FA20:1	Positive
356	902.8	577.5	TAG54:3-FA20:2	Positive
357	902.8	579.5	TAG54:3-FA20:3	Positive
358	900.8	627.5	TAG54:4-FA16:0	Positive
359	900.8	629.5	TAG54:4-FA16:1	Positive
360	900.8	599.5	TAG54:4-FA18:0	Positive
361	900.8	601.5	TAG54:4-FA18:1	Positive
362	900.8	603.5	TAG54:4-FA18:2	Positive
363	900.8	605.5	TAG54:4-FA18:3	Positive
364	900.8	573.5	TAG54:4-FA20:1	Positive
365	900.8	575.5	TAG54:4-FA20:2	Positive
366	900.8	577.5	TAG54:4-FA20:3	Positive
367	900.8	579.5	TAG54:4-FA20:4	Positive
368	900.8	545.5	TAG54:4-FA22:1	Positive
369	900.8	551.5	TAG54:4-FA22:4	Positive
370	898.8	625.5	TAG54:5-FA16:0	Positive
371	898.8	627.5	TAG54:5-FA16:1	Positive
372	898.8	597.5	TAG54:5-FA18:0	Positive
373	898.8	599.5	TAG54:5-FA18:1	Positive
374	898.8	601.5	TAG54:5-FA18:2	Positive
375	898.8	603.5	TAG54:5-FA18:3	Positive

376	898.8	573.5	TAG54:5-FA20:2	Positive
377	898.8	575.5	TAG54:5-FA20:3	Positive
378	898.8	577.5	TAG54:5-FA20:4	Positive
379	898.8	579.5	TAG54:5-FA20:5	Positive
380	898.8	543.5	TAG54:5-FA22:1	Positive
381	898.8	549.5	TAG54:5-FA22:4	Positive
382	898.8	551.5	TAG54:5-FA22:5	Positive
383	896.8	623.5	TAG54:6-FA16:0	Positive
384	896.8	625.5	TAG54:6-FA16:1	Positive
385	896.8	597.5	TAG54:6-FA18:1	Positive
386	896.8	599.5	TAG54:6-FA18:2	Positive
387	896.8	601.5	TAG54:6-FA18:3	Positive
388	896.8	573.5	TAG54:6-FA20:3	Positive
389	896.8	575.5	TAG54:6-FA20:4	Positive
390	896.8	577.5	TAG54:6-FA20:5	Positive
391	896.8	549.5	TAG54:6-FA22:5	Positive
392	896.8	551.5	TAG54:6-FA22:6	Positive
393	894.8	623.5	TAG54:7-FA16:1	Positive
394	894.8	595.5	TAG54:7-FA18:1	Positive
395	894.8	597.5	TAG54:7-FA18:2	Positive
396	894.8	599.5	TAG54:7-FA18:3	Positive
397	894.8	573.5	TAG54:7-FA20:4	Positive
398	894.8	575.5	TAG54:7-FA20:5	Positive
399	894.8	547.5	TAG54:7-FA22:5	Positive
400	894.8	549.5	TAG54:7-FA22:6	Positive
401	892.8	595.5	TAG54:8-FA18:2	Positive
402	892.8	597.5	TAG54:8-FA18:3	Positive
403	892.8	571.5	TAG54:8-FA20:4	Positive
404	892.8	573.5	TAG54:8-FA20:5	Positive
405	892.8	547.5	TAG54:8-FA22:6	Positive
406	916.7	619.4	TAG56:10-FA18:2	Positive
407	934.9	661.6	TAG56:1-FA16:0	Positive
408	934.9	635.6	TAG56:1-FA18:1	Positive
409	932.9	659.6	TAG56:2-FA16:0	Positive
410	932.9	631.6	TAG56:2-FA18:0	Positive
411	932.9	603.6	TAG56:2-FA20:0	Positive
412	932.9	605.6	TAG56:2-FA20:1	Positive
413	930.8	657.5	TAG56:3-FA16:0	Positive
414	930.8	629.5	TAG56:3-FA18:0	Positive
415	930.8	631.5	TAG56:3-FA18:1	Positive
416	930.8	633.5	TAG56:3-FA18:2	Positive
417	930.8	601.5	TAG56:3-FA20:0	Positive
418	930.8	603.5	TAG56:3-FA20:1	Positive
419	930.8	605.5	TAG56:3-FA20:2	Positive
420	928.8	655.5	TAG56:4-FA16:0	Positive
421	928.8	627.5	TAG56:4-FA18:0	Positive
422	928.8	629.5	TAG56:4-FA18:1	Positive

423	928.8	631.5	TAG56:4-FA18:2	Positive
424	928.8	601.5	TAG56:4-FA20:1	Positive
425	928.8	603.5	TAG56:4-FA20:2	Positive
426	928.8	605.5	TAG56:4-FA20:3	Positive
427	928.8	607.5	TAG56:4-FA20:4	Positive
428	928.8	579.5	TAG56:4-FA22:4	Positive
429	926.8	653.5	TAG56:5-FA16:0	Positive
430	926.8	625.5	TAG56:5-FA18:0	Positive
431	926.8	627.5	TAG56:5-FA18:1	Positive
432	926.8	629.5	TAG56:5-FA18:2	Positive
433	926.8	599.5	TAG56:5-FA20:1	Positive
434	926.8	601.5	TAG56:5-FA20:2	Positive
435	926.8	603.5	TAG56:5-FA20:3	Positive
436	926.8	605.5	TAG56:5-FA20:4	Positive
437	926.8	577.5	TAG56:5-FA22:4	Positive
438	926.8	579.5	TAG56:5-FA22:5	Positive
439	924.8	651.5	TAG56:6-FA16:0	Positive
440	924.8	623.5	TAG56:6-FA18:0	Positive
441	924.8	625.5	TAG56:6-FA18:1	Positive
442	924.8	627.5	TAG56:6-FA18:2	Positive
443	924.8	629.5	TAG56:6-FA18:3	Positive
444	924.8	599.5	TAG56:6-FA20:2	Positive
445	924.8	601.5	TAG56:6-FA20:3	Positive
446	924.8	603.5	TAG56:6-FA20:4	Positive
447	924.8	605.5	TAG56:6-FA20:5	Positive
448	924.8	575.5	TAG56:6-FA22:4	Positive
449	924.8	577.5	TAG56:6-FA22:5	Positive
450	924.8	579.5	TAG56:6-FA22:6	Positive
451	922.8	649.5	TAG56:7-FA16:0	Positive
452	922.8	651.5	TAG56:7-FA16:1	Positive
453	922.8	621.5	TAG56:7-FA18:0	Positive
454	922.8	623.5	TAG56:7-FA18:1	Positive
455	922.8	625.5	TAG56:7-FA18:2	Positive
456	922.8	627.5	TAG56:7-FA18:3	Positive
457	922.8	599.5	TAG56:7-FA20:3	Positive
458	922.8	601.5	TAG56:7-FA20:4	Positive
459	922.8	603.5	TAG56:7-FA20:5	Positive
460	922.8	573.5	TAG56:7-FA22:4	Positive
461	922.8	575.5	TAG56:7-FA22:5	Positive
462	922.8	577.5	TAG56:7-FA22:6	Positive
463	920.8	647.5	TAG56:8-FA16:0	Positive
464	920.8	649.5	TAG56:8-FA16:1	Positive
465	920.8	621.5	TAG56:8-FA18:1	Positive
466	920.8	623.5	TAG56:8-FA18:2	Positive
467	920.8	625.5	TAG56:8-FA18:3	Positive
468	920.8	599.5	TAG56:8-FA20:4	Positive
469	920.8	601.5	TAG56:8-FA20:5	Positive

470	920.8	573.5	TAG56:8-FA22:5	Positive	
471	920.8	575.5	TAG56:8-FA22:6	Positive	
472	918.8	623.5	TAG56:9-FA18:3	Positive	
473	918.8	597.5	TAG56:9-FA20:4	Positive	
474	918.8	599.5	TAG56:9-FA20:5	Positive	
475	918.8	573.5	TAG56:9-FA22:6	Positive	
476	829.8	570.56	IS_TAG	Positive	
477	806.8	264.4	dLCER(12:0)	Positive	
478	871.9	264.4	dLCER(16:0)	Positive	
479	399.262	85	Carboxytridecenoylc	Positive	
480	311.419	85	Decadienoylcarnitine	Positive	
481	315.449	85	Decanoylcarnitine_A	Positive	
482	309.401	85	Decatrienoylcarnitin	Positive	
483	313.432	85	Decenoylcarnitine_A	Positive	
484	314.5	85	Decenoylcarnitine_C	Positive	
485	203.2	70.1	Dimethylarginine	Positive	
486	329.475	85	Dimethylnonanoylca	Positive	
487	373.484	85	Dodecanedioylcarnit	Positive	
488	343.501	85	Dodecanoylcarnitine	Positive	
489	341.486	85	Dodecenoylcarnitine	Positive	
490	342.5	85	Dodecenoylcarnitine	Positive	
491	413.635	85	Heptadecanoylcarnit	Positive	
492	273.368	85	Heptanoylcarnitine_	Positive	
493	395.58	85	Hexadecadienoylcarı	Positive	
494	396.577	85	Hexadecadienyl-L-ca	Positive	
495	399.608	85	Hexadecanoylcarniti	Positive	
496	397.596	85	Hexadecenoylcarniti	Positive	
497	411.575	85	Hydroxyhexadecadie	Positive	
498	415.6	85	Hydroxyhexadecano	Positive	
499	413.595	85	Hydroxyhexadeceno	Positive	
500	387.298	85	Hydroxymyristoylcar	Positive	
501	441.648	85	Hydroxyoctadecenoy	Positive	
502	442.7	85	Hydroxyoctadecenoy	Positive	
503	301.379	85	Hydroxyoctenoylcarı	Positive	
504	383.522	85	Hydroxytetradecadie	Positive	
505	385.538	85	Hydroxytetradeceno	Positive	
506	246.1	85	Isovalerylcarnitine_C	Positive	
507	301.422	85	Nonaylcarnitine_AC(	Positive	
508	427.661	85	Octadecanoylcarnitir	Positive	
509	425.645	85	Octadecenoylcarnitir	Positive	
510	287.395	85	Octanoylcarnitine_A	Positive	
511	285.379	85	Octenoylcarnitine_A	Positive	
512	302.2	85	O-nonanoyl-L-carniti	Positive	
513	385.582	85	Pentadecanoylcarnit	Positive	
514	303.351	85	Pimeloylcarnitine_A(	Positive	
515	218.1	85	Propionylcarnitine_C	Positive	
516	262.1	84.9	Succinylcarnitine/3-h	Positive	3-hydroxyisovalerylcarnitine_C5-O

517	367.523	85	Tetradecadienoylcar	Positive
518	371.555	85	Tetradecanoylcarniti	Positive
519	369.539	85	Tetradecenoylcarniti	Positive
520	357.288	85	Tridecanoylcarnitine	Positive
521	267.2	267.2	IS_FFA_2	Negative
522	264.2	264.2	IS_FFA_1	Negative
523	199.2	199.2	FFA(12:0)	Negative
524	227.2	227.2	FFA(14:0)	Negative
525	225.2	225.2	FFA(14:1)	Negative
526	241.2	241.2	FFA(15:0)	Negative
527	255.2	255.2	FFA(16:0)	Negative
528	253.2	253.2	FFA(16:1)	Negative
529	269.2	269.2	FFA(17:0)	Negative
530	283.3	283.3	FFA(18:0)	Negative
531	281.2	281.2	FFA(18:1)	Negative
532	279.2	279.2	FFA(18:2)	Negative
533	277.2	277.2	FFA(18:3)	Negative
534	275.2	275.2	FFA(18:4)	Negative
535	311.3	311.3	FFA(20:0)	Negative
536	309.3	309.3	FFA(20:1)	Negative
537	307.3	307.3	FFA(20:2)	Negative
538	305.2	305.2	FFA(20:3)	Negative
539	303.2	303.2	FFA(20:4)	Negative
540	301.2	301.2	FFA(20:5)	Negative
541	339.3	339.3	FFA(22:0)	Negative
542	337.3	337.3	FFA(22:1)	Negative
543	335.3	335.3	FFA(22:2)	Negative
544	331.3	331.3	FFA(22:4)	Negative
545	329.2	329.2	FFA(22:5)	Negative
546	327.2	327.2	FFA(22:6)	Negative
547	367.4	367.4	FFA(24:0)	Negative
548	365.3	365.3	FFA(24:1)	Negative
549	409.2	152.8	LPA(16:0)	Negative
550	407.2	152.8	LPA(16:1)	Negative
551	437.3	152.8	LPA(18:0)	Negative
552	435.3	152.8	LPA(18:1)	Negative
553	433.2	152.8	LPA(18:2)	Negative
554	498.3	199.2	LPC(12:0)	Negative
555	526.3	227.2	LPC(14:0)	Negative
556	524.3	225.2	LPC(14:1)	Negative
557	540.3	241.2	LPC(15:0)	Negative
558	554.3	255.2	LPC(16:0)	Negative
559	552.3	253.2	LPC(16:1)	Negative
560	568.3	269.2	LPC(17:0)	Negative
561	582.4	283.3	LPC(18:0)	Negative
562	580.4	281.2	LPC(18:1)	Negative
563	578.3	279.2	LPC(18:2)	Negative

564	576.3	277.2	LPC(18:3)	Negative	
565	574.3	275.2	LPC(18:4)	Negative	
566	610.4	311.3	LPC(20:0)	Negative	
567	608.4	309.3	LPC(20:1)	Negative	
568	606.4	307.3	LPC(20:2)	Negative	
569	604.4	305.2	LPC(20:3)	Negative	
570	602.4	303.2	LPC(20:4)	Negative	
571	600.3	301.2	LPC(20:5)	Negative	
572	638.4	339.3	LPC(22:0)	Negative	
573	636.4	337.3	LPC(22:1)	Negative	
574	628.4	329.2	LPC(22:5)	Negative	
575	626.3	327.2	LPC(22:6)	Negative	
576	666.5	367.4	LPC(24:0)	Negative	
577	664.5	365.3	LPC(24:1)	Negative	
578	438.3	241.2	LPE(15:0)	Negative	
579	452.3	255.2	LPE(16:0)	Negative	
580	450.3	253.2	LPE(16:1)	Negative	
581	466.3	269.2	LPE(17:0)	Negative	
582	480.3	283.3	LPE(18:0)	Negative	
583	478.3	281.2	LPE(18:1)	Negative	
584	476.3	279.2	LPE(18:2)	Negative	
585	474.3	277.2	LPE(18:3)	Negative	
586	508.3	311.3	LPE(20:0)	Negative	
587	506.3	309.3	LPE(20:1)	Negative	
588	504.3	307.3	LPE(20:2)	Negative	
589	502.3	305.2	LPE(20:3)	Negative	
590	500.3	303.2	LPE(20:4)	Negative	
591	536.4	339.3	LPE(22:0)	Negative	
592	534.4	337.3	LPE(22:1)	Negative	
593	528.3	331.3	LPE(22:4)	Negative	
594	526.3	329.2	LPE(22:5)	Negative	
595	524.3	327.2	LPE(22:6)	Negative	
596	564.4	367.4	LPE(24:0)	Negative	
597	562.4	365.3	LPE(24:1)	Negative	
598	571.3	240.9	LPI(16:0)	Negative	
599	569.3	240.9	LPI(16:1)	Negative	
600	599.3	240.9	LPI(18:0)	Negative	
601	597.3	240.9	LPI(18:1)	Negative	
602	595.3	240.9	LPI(18:2)	Negative	
603	621.3	240.9	LPI(20:3)	Negative	
604	666.6	288.3	IS_PA_d7_(15:0/18::	Negative	18:1)
605	617.4	227.3	PA(14:0/16:1)	Negative	16:1)
606	617.4	253.3	PA(14:0/16:1)_2	Negative	16:1)_2
607	647.5	255.3	PA(16:0/16:0)	Negative	16:0)
608	645.5	253.3	PA(16:0/16:1)	Negative	16:1)
609	645.5	255.3	PA(16:0/16:1)_2	Negative	16:1)_2
610	675.5	255.3	PA(16:0/18:0)	Negative	18:0)

611	675.5	283.3	PA(16:0/18:0)_2	Negative	18:0)_2
612	673.5	255.3	PA(16:0/18:1)	Negative	18:1)
613	673.5	281.3	PA(16:0/18:1)_2	Negative	18:1)_2
614	671.5	255.3	PA(16:0/18:2)	Negative	18:2)
615	671.5	279.3	PA(16:0/18:2)_2	Negative	18:2)_2
616	697.5	255.3	PA(16:0/20:3)	Negative	20:3)
617	697.5	305.2	PA(16:0/20:3)_2	Negative	20:3)_2
618	643.4	253.3	PA(16:1/16:1)	Negative	16:1)
619	673.5	253.3	PA(16:1/18:0)	Negative	18:0)
620	673.5	283.3	PA(16:1/18:0)_2	Negative	18:0)_2
621	671.5	253.3	PA(16:1/18:1)	Negative	18:1)
622	671.5	281.3	PA(16:1/18:1)_2	Negative	18:1)_2
623	669.5	253.3	PA(16:1/18:2)	Negative	18:2)
624	669.5	279.3	PA(16:1/18:2)_2	Negative	18:2)_2
625	703.5	283.3	PA(18:0/18:0)	Negative	18:0)
626	701.5	281.3	PA(18:0/18:1)	Negative	18:1)
627	701.5	283.3	PA(18:0/18:1)_2	Negative	18:1)_2
628	699.5	279.3	PA(18:0/18:2)	Negative	18:2)
629	699.5	283.3	PA(18:0/18:2)_2	Negative	18:2)_2
630	697.5	277.3	PA(18:0/18:3)	Negative	18:3)
631	697.5	283.3	PA(18:0/18:3)_2	Negative	18:3)_2
632	725.5	283.3	PA(18:0/20:3)	Negative	20:3)
633	725.5	305.2	PA(18:0/20:3)_2	Negative	20:3)_2
634	699.5	281.3	PA(18:1/18:1)	Negative	18:1)
635	697.5	279.3	PA(18:1/18:2)	Negative	18:2)
636	697.5	281.3	PA(18:1/18:2)_2	Negative	18:2)_2
637	723.5	281.3	PA(18:1/20:3)	Negative	20:3)
638	723.5	305.2	PA(18:1/20:3)_2	Negative	20:3)_2
639	803.6	327.2	PA(22:0/22:6)	Negative	22:6)
640	803.6	339.2	PA(22:0/22:6)_2	Negative	22:6)_2
641	591.4	171.1	PA(28:0)	Negative	
642	591.4	199.2	PA(28:0)_2	Negative	
643	591.4	255.2	PA(28:0)_3	Negative	
644	591.4	283.2	PA(28:0)_4	Negative	
645	811.6	288.3	IS_PC	Negative	
646	734.5	253.2	PC(12:0/16:1)	Negative	16:1)
647	762.5	281.2	PC(12:0/18:1)	Negative	18:1)
648	760.5	279.2	PC(12:0/18:2)	Negative	18:2)
649	784.5	303.2	PC(12:0/20:4)	Negative	20:4)
650	736.5	227.2	PC(14:0/14:0)	Negative	14:0)
651	734.5	225.2	PC(14:0/14:1)	Negative	14:1)
652	762.5	253.2	PC(14:0/16:1)	Negative	16:1)
653	790.6	281.2	PC(14:0/18:1)	Negative	18:1)
654	788.5	279.2	PC(14:0/18:2)	Negative	18:2)
655	786.5	277.2	PC(14:0/18:3)	Negative	18:3)
656	818.6	309.3	PC(14:0/20:1)	Negative	20:1)
657	816.6	307.3	PC(14:0/20:2)	Negative	20:2)

658	814.6	305.2	PC(14:0/20:3)	Negative	20:3)
659	812.5	303.2	PC(14:0/20:4)	Negative	20:4)
660	810.5	301.2	PC(14:0/20:5)	Negative	20:5)
661	840.6	331.3	PC(14:0/22:4)	Negative	22:4)
662	838.6	329.2	PC(14:0/22:5)	Negative	22:5)
663	836.5	327.2	PC(14:0/22:6)	Negative	22:6)
664	776.5	241.2	PC(15:0/16:1)	Negative	16:1)
665	804.6	241.2	PC(15:0/18:1)	Negative	18:1)
666	802.6	241.2	PC(15:0/18:2)	Negative	18:2)
667	828.6	241.2	PC(15:0/20:3)	Negative	20:3)
668	826.6	241.2	PC(15:0/20:4)	Negative	20:4)
669	852.6	241.2	PC(15:0/22:5)	Negative	22:5)
670	850.6	241.2	PC(15:0/22:6)	Negative	22:6)
671	736.5	255.2	PC(16:0/12:0)	Negative	12:0)
672	764.5	227.2	PC(16:0/14:0)	Negative	14:0)
673	762.5	225.2	PC(16:0/14:1)	Negative	14:1)
674	792.6	255.2	PC(16:0/16:0)	Negative	16:0)
675	790.6	253.2	PC(16:0/16:1)	Negative	16:1)
676	820.6	283.3	PC(16:0/18:0)	Negative	18:0)
677	818.6	281.2	PC(16:0/18:1)	Negative	18:1)
678	816.6	279.2	PC(16:0/18:2)	Negative	18:2)
679	814.6	277.2	PC(16:0/18:3)	Negative	18:3)
680	812.5	275.2	PC(16:0/18:4)	Negative	18:4)
681	846.6	309.3	PC(16:0/20:1)	Negative	20:1)
682	844.6	307.3	PC(16:0/20:2)	Negative	20:2)
683	842.6	305.2	PC(16:0/20:3)	Negative	20:3)
684	840.6	303.2	PC(16:0/20:4)	Negative	20:4)
685	838.6	301.2	PC(16:0/20:5)	Negative	20:5)
686	872.6	335.3	PC(16:0/22:2)	Negative	22:2)
687	868.6	331.3	PC(16:0/22:4)	Negative	22:4)
688	866.6	329.2	PC(16:0/22:5)	Negative	22:5)
689	864.6	327.2	PC(16:0/22:6)	Negative	22:6)
690	804.6	269.2	PC(17:0/16:1)	Negative	16:1)
691	832.6	269.2	PC(17:0/18:1)	Negative	18:1)
692	830.6	269.2	PC(17:0/18:2)	Negative	18:2)
693	856.6	269.2	PC(17:0/20:3)	Negative	20:3)
694	854.6	269.2	PC(17:0/20:4)	Negative	20:4)
695	852.6	269.2	PC(17:0/20:5)	Negative	20:5)
696	882.6	269.2	PC(17:0/22:4)	Negative	22:4)
697	880.6	269.2	PC(17:0/22:5)	Negative	22:5)
698	878.6	269.2	PC(17:0/22:6)	Negative	22:6)
699	764.5	283.3	PC(18:0/12:0)	Negative	12:0)
700	792.6	227.2	PC(18:0/14:0)	Negative	14:0)
701	818.6	253.2	PC(18:0/16:1)	Negative	16:1)
702	848.6	283.3	PC(18:0/18:0)	Negative	18:0)
703	846.6	281.2	PC(18:0/18:1)	Negative	18:1)
704	844.6	279.2	PC(18:0/18:2)	Negative	18:2)

705	842.6	277.2	PC(18:0/18:3)	Negative	18:3)
706	840.6	275.2	PC(18:0/18:4)	Negative	18:4)
707	876.7	283.3	PC(18:0/20:0)	Negative	20:0)
708	874.7	309.3	PC(18:0/20:1)	Negative	20:1)
709	872.6	307.3	PC(18:0/20:2)	Negative	20:2)
710	870.6	305.2	PC(18:0/20:3)	Negative	20:3)
711	868.6	303.2	PC(18:0/20:4)	Negative	20:4)
712	866.6	301.2	PC(18:0/20:5)	Negative	20:5)
713	896.6	331.3	PC(18:0/22:4)	Negative	22:4)
714	894.6	329.2	PC(18:0/22:5)	Negative	22:5)
715	892.6	327.2	PC(18:0/22:6)	Negative	22:6)
716	788.5	225.2	PC(18:1/14:1)	Negative	14:1)
717	816.6	281.2	PC(18:1/16:1)	Negative	16:1)
718	844.6	281.2	PC(18:1/18:1)	Negative	18:1)
719	842.6	279.2	PC(18:1/18:2)	Negative	18:2)
720	840.6	277.2	PC(18:1/18:3)	Negative	18:3)
721	872.6	309.3	PC(18:1/20:1)	Negative	20:1)
722	870.6	307.3	PC(18:1/20:2)	Negative	20:2)
723	868.6	305.2	PC(18:1/20:3)	Negative	20:3)
724	866.6	303.2	PC(18:1/20:4)	Negative	20:4)
725	864.6	301.2	PC(18:1/20:5)	Negative	20:5)
726	898.7	335.3	PC(18:1/22:2)	Negative	22:2)
727	894.6	331.3	PC(18:1/22:4)	Negative	22:4)
728	892.6	329.2	PC(18:1/22:5)	Negative	22:5)
729	890.6	327.2	PC(18:1/22:6)	Negative	22:6)
730	786.5	225.2	PC(18:2/14:1)	Negative	14:1)
731	814.6	279.2	PC(18:2/16:1)	Negative	16:1)
732	840.6	279.2	PC(18:2/18:2)	Negative	18:2)
733	838.6	277.2	PC(18:2/18:3)	Negative	18:3)
734	870.6	309.3	PC(18:2/20:1)	Negative	20:1)
735	868.6	307.3	PC(18:2/20:2)	Negative	20:2)
736	866.6	305.2	PC(18:2/20:3)	Negative	20:3)
737	864.6	303.2	PC(18:2/20:4)	Negative	20:4)
738	862.6	301.2	PC(18:2/20:5)	Negative	20:5)
739	892.6	331.3	PC(18:2/22:4)	Negative	22:4)
740	890.6	329.2	PC(18:2/22:5)	Negative	22:5)
741	888.6	327.2	PC(18:2/22:6)	Negative	22:6)
742	818.6	225.2	PC(20:0/14:1)	Negative	14:1)
743	846.6	253.2	PC(20:0/16:1)	Negative	16:1)
744	874.6	281.2	PC(20:0/18:1)	Negative	18:1)
745	872.6	279.2	PC(20:0/18:2)	Negative	18:2)
746	898.7	305.2	PC(20:0/20:3)	Negative	20:3)
747	896.6	303.2	PC(20:0/20:4)	Negative	20:4)
748	894.6	301.2	PC(20:0/20:5)	Negative	20:5)
749	924.7	331.3	PC(20:0/22:4)	Negative	22:4)
750	922.7	329.2	PC(20:0/22:5)	Negative	22:5)
751	920.6	327.2	PC(20:0/22:6)	Negative	22:6)

752	709.5	288.3	IS_PE	Negative	
753	660.5	253.2	PE(14:0/16:1)	Negative	16:1)
754	688.5	281.2	PE(14:0/18:1)	Negative	18:1)
755	686.5	279.2	PE(14:0/18:2)	Negative	18:2)
756	710.5	303.2	PE(14:0/20:4)	Negative	20:4)
757	728.5	241.2	PE(15:0/20:2)	Negative	20:2)
758	750.5	241.2	PE(15:0/22:5)	Negative	22:5)
759	662.5	255.2	PE(16:0/14:0)	Negative	14:0)
760	690.5	255.2	PE(16:0/16:0)	Negative	16:0)
761	688.5	253.2	PE(16:0/16:1)	Negative	16:1)
762	716.5	281.2	PE(16:0/18:1)	Negative	18:1)
763	714.5	279.2	PE(16:0/18:2)	Negative	18:2)
764	712.5	277.2	PE(16:0/18:3)	Negative	18:3)
765	744.6	309.3	PE(16:0/20:1)	Negative	20:1)
766	742.5	307.3	PE(16:0/20:2)	Negative	20:2)
767	740.5	305.2	PE(16:0/20:3)	Negative	20:3)
768	738.5	303.2	PE(16:0/20:4)	Negative	20:4)
769	736.5	301.2	PE(16:0/20:5)	Negative	20:5)
770	766.5	331.3	PE(16:0/22:4)	Negative	22:4)
771	764.5	329.2	PE(16:0/22:5)	Negative	22:5)
772	762.5	327.2	PE(16:0/22:6)	Negative	22:6)
773	730.5	269.2	PE(17:0/18:1)	Negative	18:1)
774	728.5	269.2	PE(17:0/18:2)	Negative	18:2)
775	756.6	269.2	PE(17:0/20:2)	Negative	20:2)
776	752.5	269.2	PE(17:0/20:4)	Negative	20:4)
777	780.6	269.2	PE(17:0/22:4)	Negative	22:4)
778	778.5	269.2	PE(17:0/22:5)	Negative	22:5)
779	704.5	241.2	PE(18:0/15:0)	Negative	15:0)
780	718.5	283.3	PE(18:0/16:0)	Negative	16:0)
781	716.5	283.3	PE(18:0/16:1)	Negative	16:1)
782	746.6	283.3	PE(18:0/18:0)	Negative	18:0)
783	744.6	281.2	PE(18:0/18:1)	Negative	18:1)
784	742.5	279.2	PE(18:0/18:2)	Negative	18:2)
785	740.5	277.2	PE(18:0/18:3)	Negative	18:3)
786	772.6	309.3	PE(18:0/20:1)	Negative	20:1)
787	770.6	307.3	PE(18:0/20:2)	Negative	20:2)
788	768.6	305.2	PE(18:0/20:3)	Negative	20:3)
789	766.5	303.2	PE(18:0/20:4)	Negative	20:4)
790	764.5	301.2	PE(18:0/20:5)	Negative	20:5)
791	794.6	331.3	PE(18:0/22:4)	Negative	22:4)
792	792.6	329.2	PE(18:0/22:5)	Negative	22:5)
793	790.5	327.2	PE(18:0/22:6)	Negative	22:6)
794	714.5	281.2	PE(18:1/16:1)	Negative	16:1)
795	742.5	281.2	PE(18:1/18:1)	Negative	18:1)
796	740.5	279.2	PE(18:1/18:2)	Negative	18:2)
797	738.5	277.2	PE(18:1/18:3)	Negative	18:3)
798	770.6	309.3	PE(18:1/20:1)	Negative	20:1)

799	768.6	307.3	PE(18:1/20:2)	Negative	20:2)
800	766.5	305.2	PE(18:1/20:3)	Negative	20:3)
801	764.5	303.2	PE(18:1/20:4)	Negative	20:4)
802	762.5	301.2	PE(18:1/20:5)	Negative	20:5)
803	792.6	331.3	PE(18:1/22:4)	Negative	22:4)
804	790.5	329.2	PE(18:1/22:5)	Negative	22:5)
805	788.5	327.2	PE(18:1/22:6)	Negative	22:6)
806	712.5	279.2	PE(18:2/16:1)	Negative	16:1)
807	738.5	279.2	PE(18:2/18:2)	Negative	18:2)
808	736.5	277.2	PE(18:2/18:3)	Negative	18:3)
809	768.6	309.3	PE(18:2/20:1)	Negative	20:1)
810	766.5	307.3	PE(18:2/20:2)	Negative	20:2)
811	676.5	255.2	PE(O-16:0/16:0)	Negative	16:0)
812	674.5	253.2	PE(O-16:0/16:1)	Negative	16:1)
813	702.5	281.2	PE(O-16:0/18:1)	Negative	18:1)
814	700.5	279.2	PE(O-16:0/18:2)	Negative	18:2)
815	698.5	277.2	PE(O-16:0/18:3)	Negative	18:3)
816	726.5	305.2	PE(O-16:0/20:3)	Negative	20:3)
817	724.5	303.2	PE(O-16:0/20:4)	Negative	20:4)
818	722.5	301.2	PE(O-16:0/20:5)	Negative	20:5)
819	752.6	331.3	PE(O-16:0/22:4)	Negative	22:4)
820	750.5	329.2	PE(O-16:0/22:5)	Negative	22:5)
821	748.5	327.2	PE(O-16:0/22:6)	Negative	22:6)
822	704.6	255.2	PE(O-18:0/16:0)	Negative	16:0)
823	702.5	253.2	PE(O-18:0/16:1)	Negative	16:1)
824	730.5	281.2	PE(O-18:0/18:1)	Negative	18:1)
825	728.6	279.2	PE(O-18:0/18:2)	Negative	18:2)
826	726.5	277.2	PE(O-18:0/18:3)	Negative	18:3)
827	754.6	305.2	PE(O-18:0/20:3)	Negative	20:3)
828	752.6	303.2	PE(O-18:0/20:4)	Negative	20:4)
829	750.5	301.2	PE(O-18:0/20:5)	Negative	20:5)
830	780.6	331.3	PE(O-18:0/22:4)	Negative	22:4)
831	778.6	329.2	PE(O-18:0/22:5)	Negative	22:5)
832	776.6	327.2	PE(O-18:0/22:6)	Negative	22:6)
833	672.5	281.2	PE(P-14:0/18:1)	Negative	18:1)
834	674.5	255.2	PE(P-16:0/16:0)	Negative	16:0)
835	672.5	253.2	PE(P-16:0/16:1)	Negative	16:1)
836	702.5	283.3	PE(P-16:0/18:0)	Negative	18:0)
837	700.5	281.2	PE(P-16:0/18:1)	Negative	18:1)
838	698.5	279.2	PE(P-16:0/18:2)	Negative	18:2)
839	696.5	277.2	PE(P-16:0/18:3)	Negative	18:3)
840	728.6	309.3	PE(P-16:0/20:1)	Negative	20:1)
841	726.5	307.3	PE(P-16:0/20:2)	Negative	20:2)
842	724.5	305.2	PE(P-16:0/20:3)	Negative	20:3)
843	722.5	303.2	PE(P-16:0/20:4)	Negative	20:4)
844	720.5	301.2	PE(P-16:0/20:5)	Negative	20:5)
845	750.5	331.3	PE(P-16:0/22:4)	Negative	22:4)

846	748.5	329.2	PE(P-16:0/22:5)	Negative	22:5)
847	746.5	327.2	PE(P-16:0/22:6)	Negative	22:6)
848	702.5	255.2	PE(P-18:0/16:0)	Negative	16:0)
849	700.5	253.2	PE(P-18:0/16:1)	Negative	16:1)
850	730.6	283.3	PE(P-18:0/18:0)	Negative	18:0)
851	728.6	281.2	PE(P-18:0/18:1)	Negative	18:1)
852	726.5	279.2	PE(P-18:0/18:2)	Negative	18:2)
853	724.5	277.2	PE(P-18:0/18:3)	Negative	18:3)
854	756.6	309.3	PE(P-18:0/20:1)	Negative	20:1)
855	754.6	307.3	PE(P-18:0/20:2)	Negative	20:2)
856	752.6	305.2	PE(P-18:0/20:3)	Negative	20:3)
857	750.5	303.2	PE(P-18:0/20:4)	Negative	20:4)
858	748.5	301.2	PE(P-18:0/20:5)	Negative	20:5)
859	778.6	331.3	PE(P-18:0/22:4)	Negative	22:4)
860	776.6	329.2	PE(P-18:0/22:5)	Negative	22:5)
861	774.5	327.2	PE(P-18:0/22:6)	Negative	22:6)
862	700.5	255.2	PE(P-18:1/16:0)	Negative	16:0)
863	698.5	253.2	PE(P-18:1/16:1)	Negative	16:1)
864	726.5	281.2	PE(P-18:1/18:1)	Negative	18:1)
865	724.5	279.2	PE(P-18:1/18:2)	Negative	18:2)
866	722.5	277.2	PE(P-18:1/18:3)	Negative	18:3)
867	750.5	305.2	PE(P-18:1/20:3)	Negative	20:3)
868	748.5	303.2	PE(P-18:1/20:4)	Negative	20:4)
869	746.5	301.2	PE(P-18:1/20:5)	Negative	20:5)
870	776.6	331.3	PE(P-18:1/22:4)	Negative	22:4)
871	774.5	329.2	PE(P-18:1/22:5)	Negative	22:5)
872	772.5	327.2	PE(P-18:1/22:6)	Negative	22:6)
873	722.5	279.2	PE(P-18:2/18:2)	Negative	18:2)
874	746.5	303.2	PE(P-18:2/20:4)	Negative	20:4)
875	770.5	327.2	PE(P-18:2/22:6)	Negative	22:6)
876	740.6	288.3	IS_PG	Negative	
877	693.5	227.3	PG(14:0/16:0)	Negative	16:0)
878	693.5	255.3	PG(14:0/16:0)_2	Negative	16:0)_2
879	721.5	255.3	PG(16:0/16:0)	Negative	16:0)
880	719.5	253.3	PG(16:0/16:1)	Negative	16:1)
881	719.5	255.3	PG(16:0/16:1)_2	Negative	16:1)_2
882	749.5	255.3	PG(16:0/18:0)	Negative	18:0)
883	749.5	283.3	PG(16:0/18:0)_2	Negative	18:0)_2
884	747.5	255.3	PG(16:0/18:1)	Negative	18:1)
885	747.5	281.3	PG(16:0/18:1)_2	Negative	18:1)_2
886	745.5	255.3	PG(16:0/18:2)	Negative	18:2)
887	745.5	279.3	PG(16:0/18:2)_2	Negative	18:2)_2
888	773.5	255.3	PG(16:0/20:2)	Negative	20:2)
889	773.5	307.2	PG(16:0/20:2)_2	Negative	20:2)_2
890	771.5	255.3	PG(16:0/20:3)	Negative	20:3)
891	771.5	305.2	PG(16:0/20:3)_2	Negative	20:3)_2
892	717.5	253.3	PG(16:1/16:1)	Negative	16:1)

893	747.5	253.3	PG(16:1/18:0)	Negative	18:0)
894	747.5	283.3	PG(16:1/18:0)_2	Negative	18:0)_2
895	745.5	253.3	PG(16:1/18:1)	Negative	18:1)
896	745.5	281.3	PG(16:1/18:1)_2	Negative	18:1)_2
897	743.5	253.3	PG(16:1/18:2)	Negative	18:2)
898	743.5	279.3	PG(16:1/18:2)_2	Negative	18:2)_2
899	777.6	283.3	PG(18:0/18:0)	Negative	18:0)
900	775.5	281.3	PG(18:0/18:1)	Negative	18:1)
901	775.5	283.3	PG(18:0/18:1)_2	Negative	18:1)_2
902	773.5	279.3	PG(18:0/18:2)	Negative	18:2)
903	773.5	283.3	PG(18:0/18:2)_2	Negative	18:2)_2
904	797.5	283.3	PG(18:0/20:4)	Negative	20:4)
905	797.5	303.2	PG(18:0/20:4)_2	Negative	20:4)_2
906	773.5	281.3	PG(18:1/18:1)	Negative	18:1)
907	771.5	279.3	PG(18:1/18:2)	Negative	18:2)
908	771.5	281.3	PG(18:1/18:2)_2	Negative	18:2)_2
909	769.5	277.3	PG(18:1/18:3)	Negative	18:3)
910	769.5	281.3	PG(18:1/18:3)_2	Negative	18:3)_2
911	769.5	279.3	PG(18:2/18:2)	Negative	18:2)
912	828.6	288.3	IS_PI_d7(15:0-18:1)	Negative	
913	809.5	227.3	PI(14:0/18:0)	Negative	18:0)
914	809.5	283.3	PI(14:0/18:0)_2	Negative	18:0)_2
915	807.5	227.3	PI(14:0/18:1)	Negative	18:1)
916	807.5	281.3	PI(14:0/18:1)_2	Negative	18:1)_2
917	809.5	255.3	PI(16:0/16:0)	Negative	16:0)
918	807.5	253.3	PI(16:0/16:1)	Negative	16:1)
919	807.5	255.3	PI(16:0/16:1)_2	Negative	16:1)_2
920	837.5	255.3	PI(16:0/18:0)	Negative	18:0)
921	837.5	283.3	PI(16:0/18:0)_2	Negative	18:0)_2
922	835.5	255.3	PI(16:0/18:1)	Negative	18:1)
923	835.5	281.3	PI(16:0/18:1)_2	Negative	18:1)_2
924	833.5	255.3	PI(16:0/18:2)	Negative	18:2)
925	833.5	279.3	PI(16:0/18:2)_2	Negative	18:2)_2
926	865.6	255.3	PI(16:0/20:0)	Negative	20:0)
927	865.6	311.2	PI(16:0/20:0)_2	Negative	20:0)_2
928	863.6	255.3	PI(16:0/20:1)	Negative	20:1)
929	863.6	309.2	PI(16:0/20:1)_2	Negative	20:1)_2
930	859.5	255.3	PI(16:0/20:3)	Negative	20:3)
931	859.5	305.2	PI(16:0/20:3)_2	Negative	20:3)_2
932	857.5	255.3	PI(16:0/20:4)	Negative	20:4)
933	857.5	303.2	PI(16:0/20:4)_2	Negative	20:4)_2
934	805.5	253.3	PI(16:1/16:1)	Negative	16:1)
935	835.5	253.3	PI(16:1/18:0)	Negative	18:0)
936	835.5	283.3	PI(16:1/18:0)_2	Negative	18:0)_2
937	833.5	253.3	PI(16:1/18:1)	Negative	18:1)
938	833.5	281.3	PI(16:1/18:1)_2	Negative	18:1)_2
939	831.5	253.3	PI(16:1/18:2)	Negative	18:2)

940	831.5	279.3	PI(16:1/18:2)_2	Negative	18:2)_2
941	865.6	283.3	PI(18:0/18:0)	Negative	18:0)
942	863.6	281.3	PI(18:0/18:1)	Negative	18:1)
943	863.6	283.3	PI(18:0/18:1)_2	Negative	18:1)_2
944	861.5	279.3	PI(18:0/18:2)	Negative	18:2)
945	861.5	283.3	PI(18:0/18:2)_2	Negative	18:2)_2
946	859.5	277.3	PI(18:0/18:3)	Negative	18:3)
947	859.5	283.3	PI(18:0/18:3)_2	Negative	18:3)_2
948	889.6	283.3	PI(18:0/20:2)	Negative	20:2)
949	889.6	307.2	PI(18:0/20:2)_2	Negative	20:2)_2
950	887.6	283.3	PI(18:0/20:3)	Negative	20:3)
951	887.6	305.2	PI(18:0/20:3)_2	Negative	20:3)_2
952	885.5	283.3	PI(18:0/20:4)	Negative	20:4)
953	885.5	303.2	PI(18:0/20:4)_2	Negative	20:4)_2
954	883.5	283.3	PI(18:0/20:5)	Negative	20:5)
955	883.5	301.2	PI(18:0/20:5)_2	Negative	20:5)_2
956	913.6	283.3	PI(18:0/22:4)	Negative	22:4)
957	913.6	331.2	PI(18:0/22:4)_2	Negative	22:4)_2
958	861.5	281.3	PI(18:1/18:1)	Negative	18:1)
959	859.5	279.3	PI(18:1/18:2)	Negative	18:2)
960	859.5	281.3	PI(18:1/18:2)_2	Negative	18:2)_2
961	857.5	277.3	PI(18:1/18:3)	Negative	18:3)
962	857.5	281.3	PI(18:1/18:3)_2	Negative	18:3)_2
963	885.5	281.3	PI(18:1/20:3)	Negative	20:3)
964	885.5	305.2	PI(18:1/20:3)_2	Negative	20:3)_2
965	857.5	279.3	PI(18:2/18:2)	Negative	18:2)
966	887.6	279.3	PI(18:2/20:1)	Negative	20:1)
967	887.6	309.2	PI(18:2/20:1)_2	Negative	20:1)_2
968	915.6	305.2	PI(20:0/20:3)	Negative	20:3)
969	915.6	311.2	PI(20:0/20:3)_2	Negative	20:3)_2
970	913.6	303.2	PI(20:0/20:4)	Negative	20:4)
971	913.6	311.2	PI(20:0/20:4)_2	Negative	20:4)_2
972	719.6	288.3	IS_PS_d7(15:0/18:1)	Negative	18:1)
973	734.5	255.3	PS(16:0/16:0)	Negative	16:0)
974	732.5	253.3	PS(16:0/16:1)	Negative	16:1)
975	732.5	255.3	PS(16:0/16:1)_2	Negative	16:1)_2
976	762.5	255.3	PS(16:0/18:0)	Negative	18:0)
977	762.5	283.3	PS(16:0/18:0)_2	Negative	18:0)_2
978	760.5	255.3	PS(16:0/18:1)	Negative	18:1)
979	760.5	281.3	PS(16:0/18:1)_2	Negative	18:1)_2
980	758.5	255.3	PS(16:0/18:2)	Negative	18:2)
981	758.5	279.3	PS(16:0/18:2)_2	Negative	18:2)_2
982	784.5	255.3	PS(16:0/20:3)	Negative	20:3)
983	784.5	305.2	PS(16:0/20:3)_2	Negative	20:3)_2
984	730.5	253.3	PS(16:1/16:1)	Negative	16:1)
985	760.5	253.3	PS(16:1/18:0)	Negative	18:0)
986	760.5	283.3	PS(16:1/18:0)_2	Negative	18:0)_2

987	758.5	253.3	PS(16:1/18:1)	Negative	18:1)
988	758.5	281.3	PS(16:1/18:1)_2	Negative	18:1)_2
989	756.5	253.3	PS(16:1/18:2)	Negative	18:2)
990	756.5	279.3	PS(16:1/18:2)_2	Negative	18:2)_2
991	790.6	283.3	PS(18:0/18:0)	Negative	18:0)
992	788.5	281.3	PS(18:0/18:1)	Negative	18:1)
993	788.5	283.3	PS(18:0/18:1)_2	Negative	18:1)_2
994	786.5	279.3	PS(18:0/18:2)	Negative	18:2)
995	786.5	283.3	PS(18:0/18:2)_2	Negative	18:2)_2
996	786.5	281.3	PS(18:1/18:1)	Negative	18:1)
997	784.5	279.3	PS(18:1/18:2)	Negative	18:2)
998	784.5	281.3	PS(18:1/18:2)_2	Negative	18:2)_2
999	778.5	277.3	PS(18:3/18:3)	Negative	18:3)
1000	884.5	329.2	PS(22:4/22:5)	Negative	22:5)
1001	884.5	331.2	PS(22:4/22:5)_2	Negative	22:5)_2
1002	172.26	172.26	FFA(10:0)	Negative	
1003	186.29	186.29	FFA(11:0)	Negative	
1004	214.348	214.348	FFA(13:0)	Negative	
1005	282.461	282.461	FFA(18:1)	Negative	
1006	310.52	310.52	FFA(20:1)	Negative	
1007	338.57	338.57	FFA(22:1)_1	Negative	
1008	332.5	332.5	FFA(22:4)_1	Negative	
1009	366.62	366.62	FFA(24:1)_1	Negative	
1010	74.08	74.08	FFA(3:0)	Negative	
1011	88.11	88.11	FFA(4:0)	Negative	
1012	102.13	102.13	FFA(5:0)	Negative	
1013	116.158	116.158	FFA(6:0)	Negative	
1014	130.185	130.185	FFA(7:0)	Negative	
1015	144.21	144.21	FFA(8:0)	Negative	
1016	158.23	158.23	FFA(9:0)	Negative	
1017	578.3	279.2	LPC(18:2)	Negative	
1018	792.6	255.2	PC(32:0)_1	Negative	
1019	792.6	184	PC(32:0)_2	Negative	
1020	792.6	227.2	PC(32:0)_3	Negative	
1021	812.5	303.2	PC(34:4)_1	Negative	
1022	812.5	184	PC(34:4)_2	Negative	
1023	812.5	275.2	PC(34:4)_3	Negative	
1024	836.5	327.2	PC(36:6)_1	Negative	
1025	836.5	184	PC(36:6)_2	Negative	
1026	876.7	283.3	PC(38:0)_3	Negative	
1027	876.7	184	PC(38:0)_4	Negative	
1028	870.6	305.2	PC(38:3)_1	Negative	
1029	870.6	184	PC(38:3)_2	Negative	
1030	870.6	307.3	PC(38:3)_3	Negative	
1031	870.6	309.3	PC(38:3)_4	Negative	
1032	864.6	301.2	PC(38:6)_1	Negative	
1033	864.6	184	PC(38:6)_2	Negative	

1034	864.6	303.2	PC(38:6)_3	Negative
1035	902.6	184	PC(40:1)	Negative
1036	894.6	329.2	PC(40:5)_1	Negative
1037	894.6	184	PC(40:5)_2	Negative
1038	894.6	331.3	PC(40:5)_3	Negative
1039	894.6	301.2	PC(40:5)_4	Negative
1040	820.6	184	PC(O-34:0)	Negative
1041	840.5	184	PC(O-36:4)	Negative
1042	878.7	184	PC(O-40:6)	Negative
1043	916.7	184	PC(O-42:1)	Negative

Supplemental Table S3- Targeted Lipidomics Most Highly Changed (by Fold Change)

Top 50 features identified by fold change analysis

	Peaks(mz/rt)	Fold Change	log2(FC)
1	TAG52:4-FA20:3	2.0457	1.0326
2	Carboxytridecenoylcarnitine_AC(14:1-DC)	1.9868	0.99044
3	Hexadecanoylcarnitine_AC(16:0)	1.8885	0.91721
4	DAG(16:0/22:5)	1.8389	0.87883
5	DAG(18:2/18:3)	1.7999	0.84796
6	PC(18:1/18:3)	1.7976	0.84607
7	PC(17:0/22:5)	1.7419	0.80067
8	TAG48:0-FA16:0	1.7	0.76553
9	TAG56:9-FA20:4	1.6651	0.73562
10	TAG52:5-FA14:0	1.6613	0.73232
11	DAG(16:0/18:3)	1.6583	0.72969
12	TAG52:5-FA20:3	1.6493	0.72182
13	TAG50:5-FA16:1	1.6432	0.71653
14	TAG50:4-FA14:1	1.6271	0.7023
15	TAG48:5-FA18:3	1.6254	0.70082
16	TAG50:1-FA18:0	1.6245	0.69999
17	TAG54:5-FA22:4	1.611	0.68792
18	Hydroxyoctadecenoylcarnitine_AC(18:1-OH)	1.599	0.67714
19	DAG(14:1/18:1)	1.5934	0.67208
20	TAG52:5-FA18:1	1.5888	0.66794
21	DAG(18:1/20:5)	1.5809	0.66075
22	DAG(16:1/20:4)	1.5722	0.65274
23	PA(16:1/18:0)	1.5579	0.63961
24	PE(O-16:0/16:1)	1.5577	0.63946
25	TAG48:0-FA14:0	1.5358	0.61899
26	TAG52:6-FA20:4	1.5322	0.6156
27	TAG52:5-FA20:5	1.5296	0.61318
28	TAG48:3-FA16:1	1.5282	0.61183
29	TAG48:1-FA12:0	1.526	0.60978
30	PC(12:0/20:4)	1.5197	0.60375
31	TAG52:2-FA20:2	1.5185	0.60265
32	DAG(16:1/18:0)	1.517	0.60126
33	Hydroxytetradecadienoylcarnitine_AC(14:2-OH)	1.5034	0.5882
34	PC(18:1/22:4)	1.501	0.58593
35	TAG50:4-FA16:0	1.4867	0.57211
36	PC(16:0/22:6)	1.4705	0.5563
37	PC(16:0/18:3)	1.4673	0.55316
38	PC(14:0/16:1)	1.4628	0.54873
39	DAG(18:1/20:1)	1.459	0.54495
40	TAG48:3-FA14:1	1.456	0.54203
41	PC(16:0/14:1)	1.4557	0.54174
42	TAG52:3-FA18:0	1.4542	0.54025
43	TAG48:1-FA16:1	1.4518	0.53786
44	TAG56:8-FA20:4	1.4482	0.53423
45	TAG48:1-FA16:0	1.4481	0.53414
46	TAG52:3-FA20:2	1.4443	0.53041
47	TAG48:3-FA18:1	1.4431	0.52918
48	LPC(16:1)	1.4423	0.52835
49	PC(16:0/18:2)	1.4373	0.52336
50	TAG48:0-FA18:0	1.4339	0.51993

Supplemental Table S4- Targeted Lipidomics Most Highly Changed (by p-value)

Important features selected by t-tests with threshold 0.05. The red circles represent features above the threshold. Note the p values are transformed by -log10 so that the more significant features (with smaller p values) will be plotted higher on the graph.

Important features identified by t-tests

	Peaks(mz/rt)	t.stat	p.value	-log10(p)	FDR
1	Carboxytridecenoylcarnitine_AC(14:1-DC)	-3.6019	0.0032202	2.4921	0.62133
2	PC(18:1/18:3)	-3.3401	0.0053206	2.274	0.62133
3	Hexadecanoylcarnitine_AC(16:0)	-3.1626	0.0074873	2.1257	0.62133
4	MAG(20:3)	2.9585	0.01109	1.9551	0.62133
5	DAG(16:0/22:5)	-2.9211	0.011916	1.9239	0.62133
6	PC(16:0/18:2)	-2.8606	0.013382	1.8735	0.62133
7	Hydroxytetradecadienoylcarnitine_AC(14:2-OH)	-2.8566	0.013485	1.8701	0.62133
8	TAG56:9-FA20:4	-2.7929	0.015236	1.8171	0.62133
9	DAG(18:1/20:5)	-2.771	0.015887	1.799	0.62133
10	PG(16:1/16:1)	-2.6831	0.018791	1.726	0.62133
11	PC(17:0/22:5)	-2.6628	0.019529	1.7093	0.62133
12	PC(16:0/14:1)	-2.6267	0.020918	1.6795	0.62133
13	PA(16:1/18:0)	-2.6127	0.02148	1.668	0.62133
14	PC(15:0/18:1)	-2.5941	0.022252	1.6526	0.62133
15	PC(14:0/20:4)	-2.5774	0.022972	1.6388	0.62133
16	Hydroxyoctadecenoylcarnitine_AC(18:1-OH)	-2.5727	0.023176	1.635	0.62133
17	PC(16:0/22:6)	-2.5636	0.023577	1.6275	0.62133
18	Pentadecanoylcarnitine_AC(15:0)	-2.5011	0.026535	1.5762	0.65858
19	PE(O-16:0/16:1)	-2.4595	0.028694	1.5422	0.65858
20	PC(16:0/18:3)	-2.4003	0.032067	1.4939	0.65858
21	PC(14:0/16:1)	-2.3778	0.033447	1.4756	0.65858
22	PC(14:0/22:6)	-2.3696	0.033959	1.4691	0.65858
23	PC(12:0/20:4)	-2.3593	0.034619	1.4607	0.65858
24	LPC(16:1)	-2.3492	0.035281	1.4525	0.65858
25	PC(18:1/22:4)	-2.3047	0.038321	1.4166	0.66538
26	TAG56:8-FA20:4	-2.2894	0.039427	1.4042	0.66538
27	PC(18:0/18:0)	-2.2802	0.040101	1.3968	0.66538
28	LPC(20:4)	-2.2454	0.042769	1.3689	0.6843
29	DAG(16:1/20:4)	-2.1813	0.048125	1.3176	0.71707

Supplemental Table S5- Positive Mode Untargeted Most Highly Changed Mass-to-Charge (by Fold Change)

Top 50 features identified by fold change analysis

	Peaks(mz/rt)	Fold Change	log2(FC)
1	326.2302_3.89	0.37299	-1.4228
2	439.3389_8.25	0.39399	-1.3438
3	484.3401_7.12	0.41793	-1.2587
4	441.3541_8.83	0.42446	-1.2363
5	391.3407_8.13	0.42624	-1.2302
6	285.2861_5.07	0.43896	-1.1878
7	362.3285_8.5	0.4519	-1.1459
8	411.3087_8.46	0.45285	-1.1429
9	442.3519_8.84	0.45678	-1.1304
10	407.3147_8.02	2.1663	1.1153
11	549.3723_7.15	0.47207	-1.0829
12	371.2533_8.74	0.47386	-1.0775
13	412.3125_8.46	0.47401	-1.077
14	550.3745_7.15	0.47584	-1.0714
15	1077.4167_4.63	0.48221	-1.0523
16	361.3313_8.5	0.48305	-1.0497
17	564.3399_7.15	0.48313	-1.0495
18	411.3328_9.11	0.48556	-1.0423
19	548.3691_7.15	0.48802	-1.035
20	528.393_7.15	0.4918	-1.0238
21	373.2678_9.21	0.49203	-1.0232
22	372.3356_5.8	0.51292	-0.96319
23	332.3318_6.6	1.9457	0.96026
24	444.3665_6.59	1.9325	0.95045
25	240.2328_5.1	0.51867	-0.9471
26	527.3905_7.15	0.52172	-0.93866
27	452.3944_7.48	0.52613	-0.92651
28	576.401_7.93	0.52646	-0.92559
29	296.0642_0.36	1.8926	0.92036
30	384.3175_8.5	0.52922	-0.91806
31	526.3871_7.15	0.5301	-0.91567
32	414.3232_8.14	0.53197	-0.9106
33	413.3242_8.13	0.53275	-0.90848
34	375.3082_8.43	0.53508	-0.90217
35	383.314_8.5	0.53712	-0.89669
36	332.267_8.23	1.8564	0.89253
37	365.277_5.09	0.54001	-0.88893
38	291.6578_7.15	0.54144	-0.88514
39	371.3264_5.81	0.54181	-0.88414
40	440.3091_8.75	0.54259	-0.88206
41	282.6738_7.16	0.54446	-0.87711
42	331.2637_8.23	1.8342	0.87513
43	343.296_5.09	0.54572	-0.87377
44	292.6556_7.15	0.54693	-0.87056
45	648.2994_7.15	0.5473	-0.86959
46	435.367_8.21	0.54816	-0.86733
47	416.3353_6.03	1.8155	0.86035
48	558.386_7.01	0.553	-0.85465
49	296.2883_6.5	0.55337	-0.85369
50	351.2815_8.61	1.807	0.85362

Supplemental Table S6- Positive Mode Untargeted Most Highly Changed Mass-to-Charge (by t-test)

Important features selected by t-tests with threshold 0.05. The red circles represent features above the threshold. Note the p values are transformed by -log10 so that the more significant features (with smaller p values) will be plotted higher on the graph.

Top 50 features identified by t-tests

	Peaks(mz/rt)	t.stat	p.value	-log10(p)	FDR
1	564.3399_7.15	6.5851	1.7555e-05	4.7556	0.047871
2	523.5997_8.84	5.4497	0.00011127	3.9536	0.14254
3	240.0642_7.23	5.2502	0.00015681	3.8046	0.14254
4	332.267_8.23	-4.9371	0.00027158	3.5661	0.15265
5	331.2637_8.23	-4.8929	0.00029378	3.532	0.15265
6	838.6271_9.89	4.7391	0.00038692	3.4124	0.15265
7	576.401_7.93	4.731	0.00039267	3.406	0.15265
8	340.2864_7.74	4.6582	0.00044782	3.3489	0.15265
9	408.2903_6.68	-4.5912	0.00050577	3.296	0.15325
10	648.2994_7.15	4.4729	0.00062771	3.2022	0.16717
11	765.5981_9.95	4.4262	0.00068389	3.165	0.16717
12	550.3745_7.15	4.3646	0.000766	3.1158	0.16717
13	236.0542_0.39	4.3432	0.00079693	3.0986	0.16717
14	484.3401_7.12	4.2536	0.00094087	3.0265	0.18173
15	415.3331_8.81	-4.1747	0.0010898	2.9626	0.18173
16	420.311_7.56	4.1342	0.0011754	2.9298	0.18173
17	290.6552_7.16	4.0976	0.0012588	2.9001	0.18173
18	218.1656_7.95	4.0382	0.0014073	2.8516	0.18173
19	725.5473_9.58	4.0232	0.0014476	2.8394	0.18173
20	381.3_7.81	4.0036	0.0015019	2.8233	0.18173
21	764.5976_9.96	3.9557	0.0016436	2.7842	0.18173
22	138.1338_7.95	3.9492	0.001664	2.7788	0.18173
23	371.1021_0.42	3.9472	0.0016703	2.7772	0.18173
24	1125.8041_9.57	3.8903	0.0018599	2.7305	0.18173
25	224.1641_7.94	3.8752	0.0019138	2.7181	0.18173
26	723.541_9.58	3.842	0.0020384	2.6907	0.18173
27	702.5622_9.59	3.8371	0.0020574	2.6867	0.18173
28	292.6556_7.15	3.8368	0.0020584	2.6865	0.18173
29	278.0197_0.44	3.8168	0.0021381	2.67	0.18173
30	701.5591_9.59	3.8011	0.0022028	2.657	0.18173
31	724.5439_9.58	3.795	0.0022286	2.652	0.18173
32	549.3723_7.15	3.7948	0.0022292	2.6518	0.18173
33	641.1498_11.74	3.7737	0.0023203	2.6345	0.18173
34	570.3561_6.65	-3.771	0.0023325	2.6322	0.18173
35	521.5202_9.95	3.7405	0.002472	2.607	0.18173
36	770.5856_9.91	3.7156	0.0025916	2.5864	0.18173
37	137.1069_8.47	3.6964	0.0026886	2.5705	0.18173
38	405.3216_8.35	3.6849	0.0027482	2.561	0.18173
39	279.6479_6.4	-3.6796	0.0027761	2.5566	0.18173
40	222.158_7.95	3.6774	0.0027875	2.5548	0.18173
41	522.5975_8.84	3.6638	0.002861	2.5435	0.18173
42	433.284_6.76	3.6559	0.0029042	2.537	0.18173
43	520.5098_9.95	3.6529	0.0029212	2.5344	0.18173
44	419.2768_8.44	3.6245	0.0030839	2.5109	0.18173
45	747.6071_9.98	3.6234	0.0030906	2.51	0.18173
46	291.6578_7.15	3.6142	0.0031456	2.5023	0.18173
47	548.3691_7.15	3.6064	0.0031922	2.4959	0.18173
48	282.6738_7.16	3.5705	0.0034198	2.466	0.18173
49	397.2711_6.83	3.5307	0.0036901	2.433	0.18173
50	526.3871_7.15	3.527	0.0037164	2.4299	0.18173

Supplemental Table S7- Negative Mode Untargeted Most Highly Changed Mass-to-Charge (by Fold Change)

Top 50 features identified by fold change analysis

	Peaks(mz/rt)	Fold Change	log2(FC)
1	316.2124_3.8	0.2247	-2.1539
2	689.4938_8.68	0.25868	-1.9508
3	270.2077_6.34	0.26321	-1.9257
4	295.1775_7.36	0.26809	-1.8992
5	1096.7623_7.13	0.2858	-1.8069
6	315.1966_4.54	3.0949	1.6299
7	294.1821_7.36	0.33602	-1.5734
8	817.5478_7.38	0.3462	-1.5303
9	293.1788_7.36	0.3557	-1.4913
10	268.1483_6.23	0.36428	-1.4569
11	313.2748_8.88	0.37692	-1.4077
12	816.5431_7.38	0.38567	-1.3746
13	267.1457_6.23	0.38966	-1.3597
14	573.3312_7.61	0.40453	-1.3057
15	326.2779_8.74	0.41198	-1.2794
16	394.2652_8.74	0.41226	-1.2784
17	301.2652_8.68	0.41543	-1.2673
18	297.2431_8.15	0.41702	-1.2618
19	265.1474_6.23	0.41722	-1.2611
20	325.2745_8.74	0.41934	-1.2538
21	266.1506_6.23	0.42144	-1.2466
22	432.2215_7.17	2.3557	1.2361
23	527.3197_7.31	0.42594	-1.2313
24	480.299_9.87	0.42941	-1.2196
25	88.0398_0.43	0.43093	-1.2145
26	172.991_1.2	0.43687	-1.1947
27	377.0832_0.4	0.43836	-1.1898
28	333.207_4.53	2.258	1.1751
29	373.2956_8.43	0.4446	-1.1694
30	399.1835_8.69	0.44538	-1.1669
31	393.2624_8.74	0.45071	-1.1497
32	792.4359_6.23	0.45184	-1.1461
33	526.3155_7.31	0.45406	-1.1391
34	407.2659_9.6	0.4582	-1.126
35	300.2623_8.68	0.46015	-1.1198
36	436.2978_10.59	0.46059	-1.1185
37	333.1355_6.2	0.4639	-1.1081
38	597.2245_8.74	0.46431	-1.1068
39	917.5224_5.84	0.46488	-1.1051
40	299.2588_8.68	0.46552	-1.1031
41	766.4326_6.25	0.4669	-1.0988
42	406.263_9.61	0.46844	-1.0941
43	461.2501_8.74	0.46861	-1.0935
44	425.2579_9.65	0.4692	-1.0917
45	395.2777_9.22	0.47011	-1.0889
46	400.185_8.68	0.47083	-1.0867
47	767.4335_6.24	0.47167	-1.0841
48	433.2822_9.89	0.47404	-1.0769
49	808.4814_6.27	0.47648	-1.0695
50	561.3512_7.13	0.48163	-1.054

Supplemental Table S8- Negative Mode Untargeted Most Highly Changed Mass-to-Charge (by t-test)

Figure 3: Important features selected by t-tests with threshold 0.05. The red circles represent features above the threshold. Note the p values are transformed by -log10 so that the more significant features (with smaller p values) will be plotted higher on the graph.

Table 3: Top 50 features identified by t-tests

	Peaks(mz/rt)	t.stat	p.value	-log10(p)	FDR
1	447.3308_8.2	7.4386	4.9137e-06	5.3086	0.013051
2	544.2679_6.25	5.2782	0.0001494	3.8256	0.10134
3	430.1762_8.22	-5.0912	0.00020693	3.6842	0.10134
4	407.2659_9.6	5.082	0.00021028	3.6772	0.10134
5	352.2859_7.91	4.9564	0.00026246	3.5809	0.10134
6	408.2743_8.29	4.9021	0.00028903	3.5391	0.10134
7	610.561_10.06	4.9015	0.0002893	3.5387	0.10134
8	708.4971_9.83	-4.7756	0.0003624	3.4408	0.10134
9	436.2978_10.59	4.7319	0.00039199	3.4067	0.10134
10	686.5322_9.6	4.7156	0.0004037	3.3939	0.10134
11	406.263_9.61	4.5802	0.00051592	3.2874	0.10134
12	694.4816_9.76	-4.5395	0.00055573	3.2551	0.10134
13	718.4821_9.68	-4.5254	0.00057023	3.2439	0.10134
14	666.2708_6.84	4.5184	0.00057756	3.2384	0.10134
15	902.6828_8.51	4.5159	0.00058022	3.2364	0.10134
16	330.2516_8.22	-4.4412	0.00066526	3.177	0.10134
17	297.2431_8.15	4.4309	0.00067796	3.1688	0.10134
18	628.3364_7.13	4.4036	0.00071298	3.1469	0.10134
19	745.5491_9.59	4.3945	0.00072497	3.1397	0.10134
20	329.2482_8.22	-4.3426	0.00079781	3.0981	0.10378
21	746.5524_9.59	4.3089	0.00084908	3.0711	0.10378
22	917.5224_5.84	4.2852	0.00088727	3.0519	0.10378
23	465.2231_8.22	-4.219	0.0010034	2.9985	0.10378
24	388.8589_12.04	4.2085	0.0010232	2.9901	0.10378
25	519.2728_10.55	4.2024	0.001035	2.9851	0.10378
26	418.2959_7.56	4.1677	0.0011041	2.957	0.10378
27	404.1602_7.95	4.0868	0.0012846	2.8912	0.10378
28	429.1736_8.23	-4.0862	0.001286	2.8908	0.10378
29	503.2216_8.68	4.0522	0.0013707	2.8631	0.10378
30	582.5226_9.98	4.03	0.0014291	2.8449	0.10378
31	374.2699_7.41	4.0205	0.0014548	2.8372	0.10378
32	301.2652_8.68	4.018	0.0014617	2.8351	0.10378
33	313.2748_8.88	4.0142	0.0014723	2.832	0.10378
34	255.2326_8.42	4.0041	0.0015004	2.8238	0.10378
35	185.0215_0.42	-3.9813	0.0015664	2.8051	0.10378
36	571.2101_8.68	3.9799	0.0015704	2.804	0.10378
37	399.1835_8.69	3.9699	0.0016002	2.7958	0.10378
38	259.2428_7.95	3.9685	0.0016045	2.7947	0.10378
39	375.2729_7.42	3.9404	0.0016921	2.7716	0.10378
40	431.2914_6.75	3.9342	0.0017119	2.7665	0.10378
41	810.6122_10.02	3.9194	0.0017602	2.7544	0.10378
42	214.0486_0.4	-3.9064	0.0018042	2.7437	0.10378
43	689.4938_8.68	3.8894	0.0018633	2.7297	0.10378
44	326.2701_7.77	3.8867	0.0018726	2.7276	0.10378
45	360.2541_7.24	3.8742	0.0019176	2.7172	0.10378
46	584.5245_9.97	3.8691	0.001936	2.7131	0.10378
47	355.1579_8.42	3.8647	0.0019522	2.7095	0.10378
48	338.2698_7.73	3.8554	0.001987	2.7018	0.10378
49	584.3913_7.41	3.8501	0.0020073	2.6974	0.10378
50	377.0832_0.4	3.8373	0.0020566	2.6869	0.10378
